# Genome-wide identification of the aldo-keto reductase gene family and functional characterization of *CsAKRIVB2* conferring cold tolerance in tea plant (*Camellia sinensis*)

**DOI:** 10.3389/fpls.2026.1950368

**Published:** 2026-09-24

**Authors:** Dandan Liu, Leigang Wang, Xiaoyu Jiao, Minghui Sun, Xu Ruan, Qian Wang, Wenjie Wang, Qiong Wu

**Affiliations:** 1 Tea Research Institute, Anhui Academy of Agricultural Sciences, Hefei, Anhui, China; 2 State Key Laboratory of Tea Plant Biology and Utilization, Anhui Agricultural University, Hefei, China

**Keywords:** Aldo-keto reductase (AKR), *Camellia sinensis*, CBF-COR signaling, cold stress tolerance, reactive oxygen species (ROS)

## Abstract

Aldo-keto reductases (AKRs) are a superfamily of NADPH-dependent oxidoreductases that play a crucial role in detoxifying reactive carbonyl species and maintaining redox homeostasis under abiotic stresses. However, systematic characterization of AKRs and their functional roles in cold stress responses in tea plants (*Camellia sinensis*) remain largely unexplored. We identified 48 *CsAKR* genes genome-wide and classified them into four groups. Structural and promoter analyses pointed to conserved protein backbones alongside diversified regulatory architectures. Expression profiling and RT-qPCR screening identified *CsAKRIVB2* as a cold-inducible candidate, which contains seven ARE motifs, the highest counts in the family. The protein localized to both the nucleus and cytoplasm. *CsAKRIVB2* overexpression consistently enhanced cold tolerance in *E. coli*, *N. benthamiana*, and transgenic *Arabidopsis*. Transgenic OE lines exhibited lower ROS and MDA levels, higher SOD/POD/CAT activities, and sustained expression of multiple cold-responsive genes, including *COR15A*, *COR15B*, *RD29A*, *KIN1*, and *SnRK2.4*, alongside upregulated antioxidant genes such as *CAT1*, *APX2*, *FSD1*, *MDAR1*, and *GR2*, under cold stress. These results demonstrate that *CsAKRIVB2* functions as a positive regulator of cold tolerance and represents a promising candidate for breeding cold-tolerant tea varieties.

## 1 Introduction

Cold stress restricts the geographic distribution of plant species and reduces global crop yields (Soualiou et al., 2022). It disrupts membrane integrity (Thomashow, 1999), osmotic homeostasis (Hare et al., 1998), and photosynthesis (Baker, 2008), while also triggering excessive accumulation of reactive oxygen species (ROS), including superoxide anion (O_2_^-^) and hydrogen peroxide (H_2_O_2_). These ROS damage membrane lipids, proteins, and nucleic acids, leading to lipid peroxidation and cell death (Gill and Tuteja, 2010; You and Chan, 2015). At low concentrations, ROS act as signaling molecules that activate stress-responsive genes, yet excess ROS cause oxidative damage under severe stress (Mittler, 2017; Baxter et al., 2014). Maintaining ROS homeostasis is thus critical for plant survival under cold conditions. Plants have evolved both enzymatic and non-enzymatic antioxidant systems to scavenge excess ROS (Ahmad et al., 2010; Gill and Tuteja, 2010), with superoxide dismutase (SOD), peroxidase (POD), and catalase (CAT) serving as primary enzymes (Gill and Tuteja, 2010; Rao et al., 2025). However, these defenses often fail under prolonged cold stress. Secondary toxic products, especially reactive carbonyl species (RCS) generated from lipid peroxidation, then require specialized detoxification mechanisms to preserve cellular integrity (Mano, 2012).

The aldo-keto reductase (AKR) superfamily comprises NADPH(nicotinamide adenine dinucleotide phosphate)-dependent oxidoreductases that reduce carbonyl substrates to alcohols (Penning, 2015). Present across prokaryotes and eukaryotes, AKRs detoxify reactive carbonyl species (RCS), such as malondialdehyde (MDA) and 4-hydroxynonenal (HNE), generated during lipid peroxidation under oxidative stress (Sengupta et al., 2015). Together with aldehyde dehydrogenases and glutathione transferases, AKRs form a cellular detoxification network that protects plants against RCS (Yalçinkaya et al., 2019). Accumulating evidence indicates that AKRs contribute to abiotic stress tolerance. Genome-wide analyses have identified 30 AKR genes in *Medicago truncatula* (Yu et al., 2020), 28 in tomato (Guan et al., 2023), and 38 in sweet cherry (Guo et al., 2025), with several showing responsiveness to salt, drought, and abscisic acid (ABA). In rice, the AKR gene *OsKOB1* is upregulated under salt and drought stress, and mutant seeds show reduced osmotic tolerance and delayed germination (Tian et al., 2025). Despite these findings, the functions of AKR genes in cold tolerance remain largely unexplored across species. This gap is particularly significant for cold-sensitive perennial crops like the tea plant.

Tea (*Camellia sinensis* (L.) O. Ktze) is a perennial beverage crop cultivated worldwide. As a subtropical evergreen, it is sensitive to low temperatures, and late spring frosts often damage tender shoots, reducing yield and quality (Li et al., 2018; Wang et al., 2022). Current cold-tolerance research in tea has focused mainly on the *CBF*/*DREB* pathway (Ding et al., 2022; Li et al., 2025) and downstream cold-responsive genes (Yin et al., 2016; Dong et al., 2025). Much less is known about detoxification mechanisms that alleviate cold-induced oxidative damage, especially the elimination of toxic aldehydes from lipid peroxidation. A systematic genome-wide characterization of the AKR gene family in *C. sinensis* is needed to clarify the roles of *CsAKR* genes under cold stress.

In this study, we performed a genome-wide identification of AKR genes in the tea plant and characterized their phylogenetic relationships, gene structures, conserved motifs, and promoter *cis*-elements. Transcriptome profiling and RT-qPCR screening identified *CsAKRIVB2* as a cold-responsive candidate, with its promoter enriched in ARE motifs. We assessed its function through heterologous expression in *Escherichia coli*, transient overexpression in *Nicotiana benthamiana*, and stable overexpression in transgenic *Arabidopsis thaliana*. Our results demonstrate that *CsAKRIVB2* acts as a positive regulator of cold tolerance, providing insights into the role of AKR genes in plant stress adaptation and identifying a candidate for breeding cold-tolerant tea varieties.

## 2 Materials and methods

### 2.1 Genome-wide identification and physicochemical characterization of *CsAKR* genes

The HMM profile of the Aldo_ket_red domain (PF00248) was retrieved from the Pfam database (http://pfam.xfam.org/) (Gulbis et al., 2000) and used as a query to search against the *C. sinensis* ‘Shuchazao’ genome using TBtools V2.110 (Chen et al., 2023), with an E-value threshold of < 1 × 10^-5^, a domain E-value threshold of < 1 × 10^-5^, and a minimum sequence length of 200 amino acids. Candidate sequences were validated using Pfam and NCBI CDD (https://www.ncbi.nlm.nih.gov/cdd). Following the criteria of Krishnamurthy et al. (2022), 48 non-redundant *CsAKR* genes were identified, renamed, and classified into four groups. Physicochemical properties were predicted using ExPASy (http://web.expasy.org/protparam/) (**Supplementary Table S1**). Chromosomal locations were extracted from the genome annotation file (GFF3) and visualized using TBtools. The protein sequences of all identified CsAKR proteins are listed in **Supplementary Table S2**.

### 2.2 Phylogenetic analysis

AKR protein sequences from *Arabidopsis thaliana* (22) and *Oryza sativa* (28) were downloaded from the Phytozome database (https://phytozome-next.jgi.doe.gov) (Goodstein et al., 2012). A neighbor-joining phylogenetic tree was constructed using MEGA7 (Kumar et al., 2016) with the JTT model and 1,000 bootstrap replicates.

### 2.3 Gene structure, conserved motif, and *cis*-acting element analysis

Conserved motifs were identified using MEME (https://meme-suite.org/meme/), with the maximum number of motifs set to 12. Exon–intron structures were extracted from the GFF3 file and visualized using TBtools. The 2,000 bp promoter sequences upstream of the translation start site of all *CsAKR* genes were submitted to PlantCARE (http://bioinformatics.psb.ugent.be/webtools/plantcare/html/) to predict stress-responsive *cis*-acting regulatory elements. The raw annotations comprised 29 distinct element labels, which were consolidated into 19 major stress-related categories according to standard nomenclature. Synonymous labels from different species-specific libraries were merged into a single category (e.g., DRE (Dehydration Responsive Element), comprising DRE core and DRE1; MYC (Myelocytomatosis related element), comprising MYC and Myc; W-BOX (WRKY binding site, W box), comprising W box; and MYB (Myeloblastosis-related element), comprising MYB, Myb, MYB recognition site, MYB-like sequence, and Myb-binding site). For each gene and each element category, the motif count was obtained. To avoid double-counting, a single physical binding site annotated under multiple synonymous labels was counted only once: two records were treated as duplicates when they shared identical sequence, position, motif length, and strand. Stress-responsive *cis*-elements were visualized using TBtools.

### 2.4 Synteny and *Ka/Ks* analysis

Intraspecific (C. *sinensis*–C. *sinensis*) and interspecific (C. *sinensis*–*C. oleifera*, C. *sinensis*–*C. chekiangoleosa*) synteny analyses were performed using MCScanX within the TBtools suite (Chen et al., 2023). *Ka/Ks* (nonsynonymous substitution rate/synonymous substitution rate) ratios and *Ks* values were calculated using the Simple *Ka/Ks* Calculator (NG). Divergence time was estimated as T = *Ks*/2λ, with λ = 6.5 × 10^-9^ (Wu et al., 2022).

### 2.5 Transcriptome data analysis

Public transcriptome datasets were obtained from the Tea Plant Information Archive (TPIA) (https://tpia.teaplants.cn/) (Gao et al., 2024), including tissue-specific profiles (Wei et al., 2018), cold acclimation and de-acclimation profiles (Wang et al., 2013), and salt and drought stress profiles (Zhang et al., 2017). Gene expression levels were quantified as FPKM (fragments per kilobase of transcript per million mapped reads). For clustering analysis, genes with FPKM values below 10 across all samples were excluded to remove low-abundance transcripts. The remaining FPKM values were transformed using log_2_(FPKM + 1) and then subjected to Z-score normalization by rows. Heatmaps were generated using TBtools (Chen et al., 2023).

### 2.6 Plant materials and stress treatments

*Camellia sinensis* cv. ‘Shuchazao’ cutting seedlings (two-year-old) were grown at 25 °C under a 16 h light/8 h dark photoperiod. For cold treatment, current-year branches were placed at 4 ± 1 °C, and the second leaf was sampled at 0, 4, 8, 12, 24, and 48 h, immediately frozen in liquid nitrogen, and stored at –80 °C.

*Arabidopsis thaliana* (Col-0) seeds were surface-sterilized, sown on 1/2 MS medium, stratified at 4 °C for 2 days, and then transferred to a growth chamber (22 °C, 16 h light/8 h dark). Seven-day-old seedlings were transplanted into nutrient soil. For freezing treatment, four-week-old seedlings were subjected to –20 °C for 2 h, followed by 24 h recovery at 22 °C. For cold stress treatment, four-week-old seedlings were incubated at 4 °C. These two treatments were used to assess cold acclimation responses and whole-plant freezing survival, respectively. *Nicotiana benthamiana* plants were grown under the same conditions as the tea plants.

### 2.7 RNA extraction and RT-qPCR

Total RNA was extracted using the RNAprep Pure Plant Plus Kit (TIANGEN, Beijing, China). First-strand cDNA was synthesized using the PrimeScript™ RT reagent Kit with gDNA Eraser (TaKaRa, Dalian, China). RT-qPCR was performed on a LightCycler^®^ 96 system (Roche, Switzerland) using SYBR^®^ Green Premix Pro Taq HS qPCR Kit (Accurate, Hunan, China). The 20-μL reaction mixture contained 10 μL of 2× SYBR Green Premix, 0.8 μL each of forward and reverse primers (10 μM), 2 μL of cDNA template, and 7.2 μL of RNase-free water. The PCR cycling program was: initial denaturation at 95 °C for 30 s, followed by 40 cycles of 95 °C for 10 s and 60 °C for 30 s. Melting curve analysis was performed under the following conditions: 95 °C for 10 s, 65 °C for 60 s, and 97 °C for 1 s to verify the specificity of each primer pair. Primers used for qPCR are listed in **Supplementary Table S8**. *CsActin* and *AtUBQ10* served as internal reference genes for tea plant and *Arabidopsis*, respectively. Relative expression levels were calculated using the 2^^–ΔΔCt^ method (Schmittgen and Livak, 2008). For transgene expression analysis, WT *Arabidopsis* was included as a negative control; any amplification with Ct > 35 cycles was considered undetectable. For comparison among transgenic lines, relative expression levels were normalized to *AtUBQ10* and then expressed relative to the OE1 line, which was set to 1.0. Each sample included three biological replicates, with three technical replicates per biological replicate.

### 2.8 Cloning of *CsAKRIVB2* and vector construction

The coding sequence (CDS)(coding sequence) of *CsAKRIVB2* (CSS0020849.1) was amplified from ‘Shuchazao’ leaf cDNA using KOD One™ PCR Master Mix (TOYOBO, Japan). For subcellular localization, the CDS (without the stop codon) was cloned into the pCAMBIA1305-*GFP* vector to generate the 35S::*CsAKRIVB2*-*GFP* construct. For overexpression in *A. thaliana*, the CDS was cloned into pCAMBIA3301 carrying a *GUS* reporter gene to generate 35S::*CsAKRIVB2*. For prokaryotic expression, the CDS was cloned into pGEX-4T-1. All constructs were verified by Sanger sequencing. Primers used for vector construction are listed in **Supplementary Table S8**.

### 2.9 Subcellular localization of *CsAKRIVB2*

The 35S::*CsAKRIVB2*-*GFP* and 35S::*GFP* constructs were transformed into *Agrobacterium tumefaciens* GV3101 and infiltrated into *Nicotiana benthamiana* leaves. At 48–72 h after infiltration, GFP fluorescence (green channel) and mCherry nuclear marker fluorescence (red channel) were visualized using a confocal laser scanning microscope. For independent confirmation of nuclear localization, leaf tissues were infiltrated with 4′,6-diamidino-2-phenylindole (DAPI, 10 μg/mL) using a syringe with 2–3 cycles of vacuum application and release, followed by incubation at room temperature in the dark for 5–10 min. DAPI fluorescence was visualized in the blue channel under the same confocal microscope.

### 2.10 Transient overexpression in *N. benthamiana*

The 35S::*CsAKRIVB2* and 35S::00 (empty vector) constructs were transformed into *A. tumefaciens* GV3101 and infiltrated into *N. benthamiana* leaves. At 2 days post-infiltration (dpi), plants were subjected to cold treatment at 4 °C for 4 h, while control plants were maintained under normal growth conditions. Leaves were then collected for nitroblue tetrazolium (NBT) and 3,3’-diaminobenzidine (DAB) staining to detect superoxide anion and hydrogen peroxide accumulation, respectively.

### 2.11 Heterologous expression in *E. coli*

The pGEX-4T-1-*CsAKRIVB2* and pGEX-4T-1 (empty vector) constructs were transformed into *E. coli* BL21(DE3). Protein expression was induced with 0.5 mM β-D-1-thiogalactopyranoside (IPTG) at 17 °C for 16 h and verified by sodium dodecyl sulfate-polyacrylamide gel electrophoresis (SDS-PAGE). Spot assays and growth curve analyses were performed as described previously (Cao et al., 2022) with minor modifications.

### 2.12 Generation of transgenic *Arabidopsis* lines

The *CsAKRIVB2* coding sequence was cloned into the pCAMBIA3301 vector under the control of the CaMV 35S promoter to generate the 35S::*CsAKRIVB2* construct. This vector carries the *bar* gene as a selectable marker conferring resistance to glufosinate ammonium for screening transgenic lines, and the *gus* reporter gene for histochemical GUS staining to qualitatively confirm transgene expression. The construct was verified by Sanger sequencing and then introduced into *A. thaliana* (Col-0) via *A. tumefaciens*-mediated floral dip transformation. Transgenic lines were screened on 1/2 MS medium containing 25 mg/L glufosinate ammonium. T_3_ homozygous lines were confirmed by GUS staining and RT-qPCR. Six T_3_ homozygous lines were obtained, and RT-qPCR confirmed *CsAKRIVB2* transcript accumulation in all transgenic lines, whereas no specific amplification was detected in WT (wild-type) plants (Ct > 35 cycles, considered undetectable). Among the six lines, OE1 and OE9 were selected as representative lines with moderate and high transgene expression levels, respectively. For comparisons among transgenic lines, the expression level of OE1 was set to 1.0.

### 2.13 Physiological and biochemical assays

Relative electrolyte leakage was measured as described (Li et al., 2019). NBT and DAB staining were performed to detect O_2_^-^ and H_2_O_2_, respectively. MDA, H_2_O_2_, O_2_^-^, SOD, POD, and CAT were measured using commercial kits (Solarbio, Beijing, China; **Supplementary Table S9**).

### 2.14 Statistical analysis

All experiments were performed with at least three biological replicates. Data are presented as means ± standard deviation (SD). Statistical significance was determined using Student’s *t*-test (for pairwise comparisons) or one-way ANOVA (for multiple comparisons). Asterisks indicate statistically significant differences (*P < 0.05, **P < 0.01, ***P < 0.001).

## 3 Results

### 3.1 Genome-wide identification and chromosomal distribution of *CsAKR* genes

Using the PF00248 hidden Markov model profile, we identified 48 putative *CsAKR* genes from the *Camellia sinensis* genome. All candidates carried the conserved Aldo_ket_red domain (PF00248), as confirmed by Pfam and NCBI CDD. Following the nomenclature of Krishnamurthy et al. (2022), the 48 *CsAKR* genes fell into four groups (Groups I–IV), with 5, 2, 2, and 5 subfamilies, respectively. Group I was the largest, comprising 25 members in five subfamilies (IA–IE). Groups II and III each had only two members. The predicted CsAKR proteins ranged from 303 to 388 amino acids, with molecular weights of 34.10–43.75 kDa and isoelectric points (pI) of 5.01–9.38. **Supplementary Table S1** lists detailed information for all *CsAKR* genes. These genes were unevenly distributed across 11 chromosomes. Chromosome 5 harbored the largest cluster, with 16 genes, whereas six genes mapped to unassembled contigs (**Supplementary Figure S1**).

### 3.2 Phylogenetic analysis

To investigate the phylogenetic relationships of the *CsAKR* family, we constructed a neighbor-joining tree using AKR protein sequences from *C. sinensis* (48), *A. thaliana* (22), and *O. sativa* (28). The 98 AKR proteins clustered into four distinct groups (Groups I–IV), with members from different species intermingled within each group (**Figure 1**). Group I was the largest and contained five subfamilies (IA–IE). Most *CsAKRIA* members formed a cluster with several rice AKRs, suggesting that this subfamily predates the divergence of monocots and dicots. Group IV exhibited the widest distribution across the phylogenetic tree, with five subfamilies (IVA, IVB, IVD, IVE, and IVF). Within Group IV, the *CsAKRIVB* subfamily (*CsAKRIVB1*, *CsAKRIVB2*, and *CsAKRIVB3*) formed a strongly supported monophyletic clade with *Arabidopsis AT2G37760.2* and *AT2G37770.2*, indicating a common ancestral origin. *CsAKRIVD1* and *CsAKRIVD2* sat extremely close on the tree, implying a recent duplication event, consistent with the segmental duplication identified in the synteny analysis (**Figure 2A**). Multiple *CsAKRIVF* members clustered with rice AKRs, suggesting possible orthology between the two species. Overall, the phylogenetic analysis revealed distinct subfamily divergence within the *CsAKR* family, reflecting a coexistence of functional conservation and diversification.

**Figure 1:**
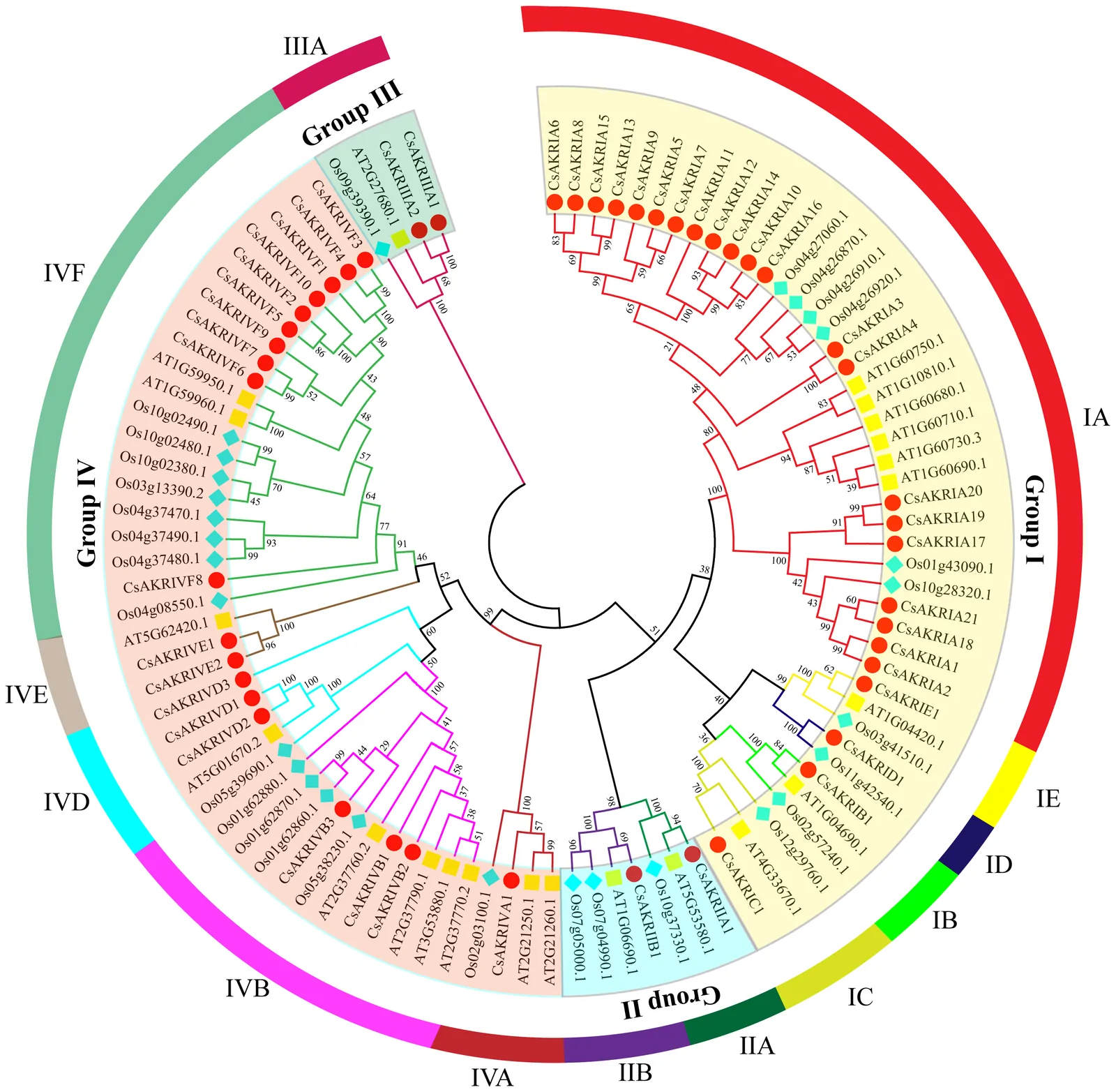
Phylogenetic analysis of CsAKR proteins. The neighbor-joining (NJ) phylogenetic tree was constructed using MEGA7 with the JTT model and 1,000 bootstrap replicates. AKR protein sequences from *C. sinensis* (48, red circles), *A. thaliana* (22, yellow squares), and *O. sativa* (28, blue diamonds) were used. Bootstrap values (1,000 replicates) are shown at branch nodes. The four major groups (I–IV) are highlighted in yellow, blue, green, and pink, respectively. Colored arcs in the outer ring indicate subfamily assignments within each group (IA-IE, IIA-IIB, IIIA,IVA-IVB and IVD-IVF).

**Figure 2:**
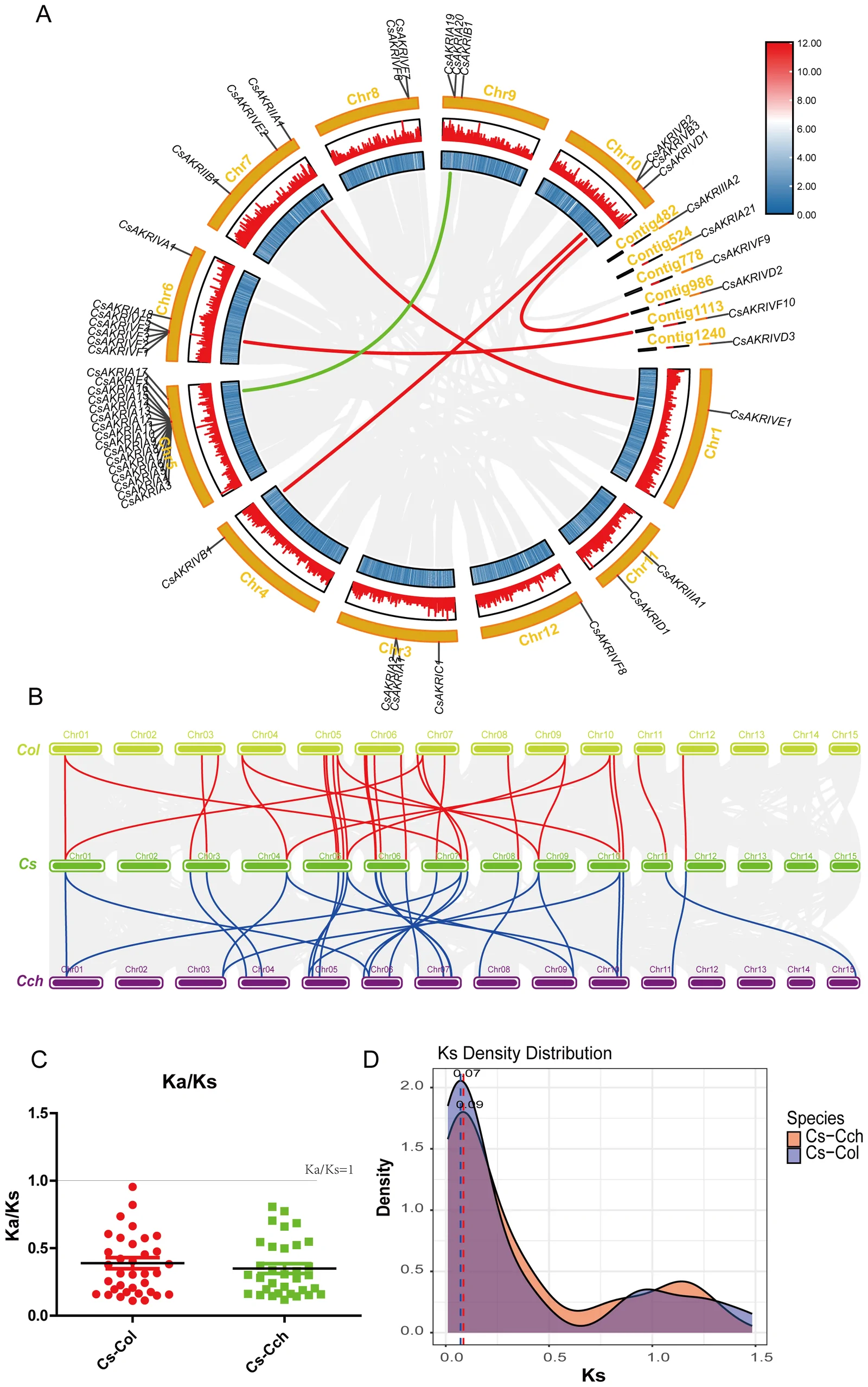
Synteny and *Ka/Ks* analysis of *AKR* genes within and among *Camellia* species. **(A)** Intraspecific synteny of *CsAKR* genes within the *C. sinensis* genome. From outer to inner: yellow circle, chromosomes with *CsAKR* positions; histogram, gene density; heatmap (blue to red), gene density gradient. Gray lines: other syntenic pairs; green and red lines: duplicated *AKR* pairs (IA and IV subfamilies, respectively). **(B)** Interspecific synteny between *C. sinensis* and two related *Camellia* species: *C. oleifera* (Cs–Col) and *C. chekiangoleosa* (Cs–Cch). Orthologous gene pairs of Cs–Col and Cs–Cch are connected by red and blue lines, respectively. **(C)** *Ka/Ks* ratios of orthologous *AKR* gene pairs between the three *Camellia* species; dashed line, *Ka/Ks* = 1. **(D)** *Ks* density distribution of orthologous *AKR* gene pairs; dashed vertical lines indicate peak *Ks* values (Cs-Col ~0.07; Cs-Cch ~0.09). *Ka/Ks* and *Ks* values were calculated using TBtools.

### 3.3 Gene structure, conserved motifs, and conserved domain analysis

To validate the phylogenetic classification, we examined the gene structures, conserved motifs, and conserved domains of *CsAKR* members. Twelve conserved motifs were identified, among which Motifs 1, 2, 3, 4, and 7 corresponded to the Aldo_ket_red domain (**Supplementary Table S3**). Members within the same group shared similar motif compositions, whereas substantial variation existed between groups (**Figures 3A, B**). Group I members contained two to four Aldo_ket_red motifs, with subfamily IA possessing the highest number of motifs. Groups II and III, which were phylogenetically close, showed similar motif patterns. Group IV members harbored two to three Aldo_ket_red motifs. Conserved domain analysis confirmed that all CsAKR proteins carried the Aldo_ket_red domain (**Figure 3C**).

**Figure 3:**
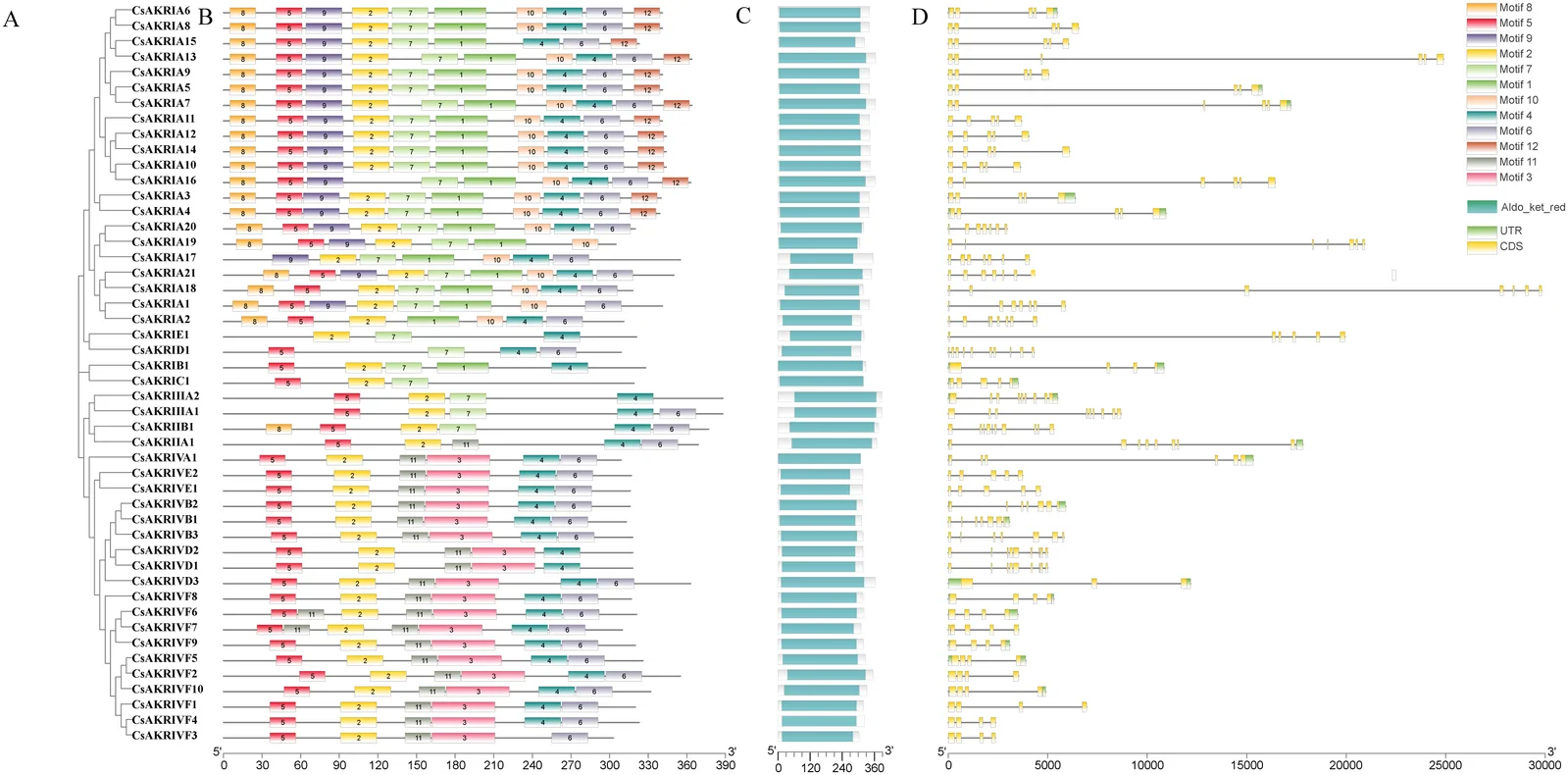
Gene structure, conserved motifs, and conserved domain analysis of CsAKR proteins. **(A)** Phylogenetic tree of CsAKR proteins. **(B)** Distribution of 12 conserved motifs identified by MEME. Different colored boxes represent different motifs. **(C)** Conserved Aldo_ket_red (PF00248) domain structure of CsAKR proteins. **(D)** Exon–intron structure of *CsAKR* genes. Exons and introns are represented by yellow boxes and black lines, respectively.

Exon–intron structures varied considerably among *CsAKR* genes (**Figure 3D**). Exon numbers ranged from 3 to 10. Group I members generally contained 4–10 exons, whereas Group IV members displayed more compact structures (3–9 exons). Detailed exon counts for each *CsAKR* gene are provided in **Supplementary Table S4**. Collectively, the conserved motif patterns and gene structures supported the phylogenetic groupings. The consistent presence of the Aldo_ket_red domain across all members confirmed them as AKR family members.

### 3.4 *Cis*-acting regulatory element analysis

To explore the regulatory mechanisms of *CsAKR* genes, we analyzed *cis*-acting elements in the 2,000 bp promoter regions upstream of the start codon using PlantCARE. A variety of hormone-responsive, stress-responsive, and development-related elements were identified across the *CsAKR* promoters (**Figure 4**; **Supplementary Table S5**). Among hormone-responsive elements, the ethylene-responsive element (ERE) and the abscisic acid-responsive element (ABRE) were the most prevalent, present in 42 and 35 *CsAKR* promoters, respectively. Methyl jasmonate-, salicylic acid-, and gibberellin-responsive elements were also detected, suggesting regulation by multiple hormone signaling pathways. For stress-responsive elements, the anaerobic responsive element (ARE) was the most widespread, found in 44 of the 48 *CsAKR* promoters. The *CsAKRIVB2* promoter contained seven ARE motifs, the highest among all family members. The low-temperature-responsive (LTR) element, a *cis*-element directly associated with low-temperature responsiveness, was detected in 13 of the 48 *CsAKR* promoters. Notably, although *CsAKRIVB2* was the most strongly cold-induced gene in our expression profiling, its promoter lacked an LTR element; by contrast, several other cold-responsive genes carried LTRs, including *CsAKRIVF9* (two LTR motifs). The drought-inducible MYB-binding site (MBS) and defense/stress-responsive (TC-rich repeats) elements were also identified in subsets of *CsAKR* promoters. The enrichment of hormone- and stress-related *cis*-elements provides a transcriptional basis for the potential involvement of *CsAKR* genes in abiotic stress responses.

**Figure 4:**
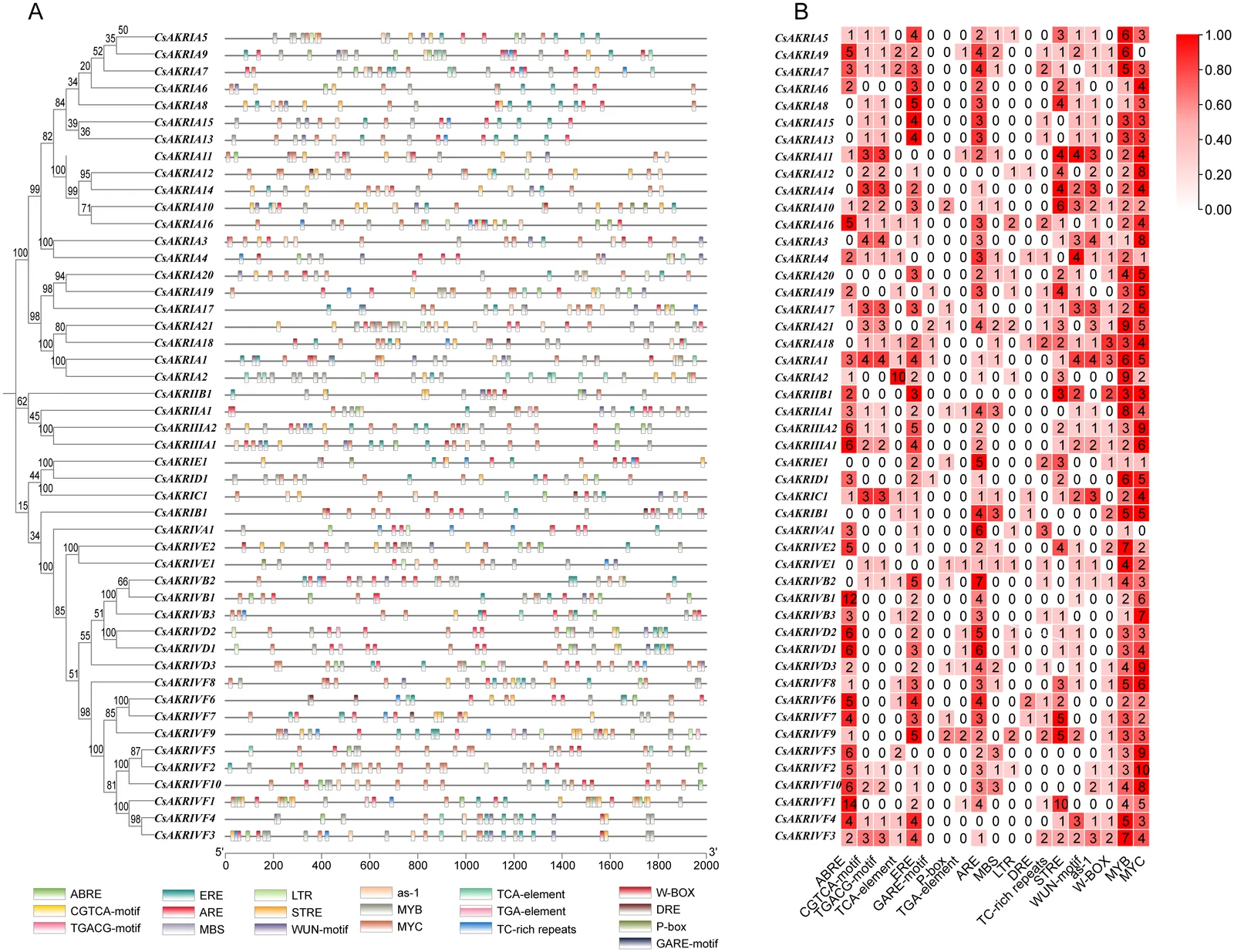
*Cis*-acting element analysis of *CsAKR* promoters. **(A)**Phylogenetic tree and promoter *cis*-element distribution. Left panel:neighbor-joining (NJ) phylogenetic tree of CsAKR proteins constructed using MEGA7 with the JTT model and 1,000 bootstrap replicates; bootstrap values are shown at branch nodes. Right panel: schematic diagram of key stress-responsive *cis*-elements in the 2,000-bp promoter regions of *CsAKR* genes, with genes arranged in the same order as the phylogenetic tree (left panel). Different colored boxes represent different types of *cis*-elements; color-coded definitions are provided below panel **(A, B)** Heatmap of *cis*-element distribution. Heatmap showing the distribution and abundance of *cis*-acting elements in the promoter regions, with genes arranged in the same order as in **(A)**. Color intensity from white to red represents element copy number from low to high, as indicated by the scale bar. The complete inventory of stress-responsive *cis*-acting elements, including motif sequence, position and strand in each promoter, is provided in **Supplementary Table S5**. Promoter sequences (2,000 bp upstream of the start codon) were analyzed using the PlantCARE database.

### 3.5 Synteny and *Ka/Ks* analysis of *CsAKR* genes

To investigate the evolutionary mechanisms underlying *CsAKR* gene family expansion, we performed intraspecific and interspecific synteny analyses. Five paralogous gene pairs, all representing segmental duplications, were identified within the *C. sinensis* genome (**Figure 2A**): *CsAKRIA17*/*CsAKRIA19*, *CsAKRIVE1*/*CsAKRIVE2*, *CsAKRIVB1*/*CsAKRIVB2*, *CsAKRIVD1*/*CsAKRIVD2*, and *CsAKRIVF5*/*CsAKRIVF10*, indicating that gene duplication contributed to family expansion. Of these, one pair (*CsAKRIA17*/*CsAKRIA19*) belongs to the IA subfamily (green line in **Figure 2A**), whereas the remaining four pairs belong to the IV subfamily (red lines). The *CsAKRIVD1*/*CsAKRIVD2* pair corroborates their close phylogenetic proximity (**Figure 1**), where they formed a highly supported sister group. Interspecific synteny analysis identified 35 orthologous *AKR* gene pairs between *C. sinensis* and *C. oleifera* (Cs–Col) and 33 between *C. sinensis* and *C. chekiangoleosa* (Cs–Cch) (**Figure 2B**). All 68 orthologous pairs had *Ka/Ks* ratios below 1 (**Figure 2C**), indicating purifying selection as the predominant force maintaining *AKR* gene function across these species. *Ks* density distribution peaks occurred at 0.07 for Cs–Col and 0.09 for Cs–Cch (**Figure 2D**; **Supplementary Table S6**), corresponding to estimated divergence times of approximately 5.39 and 6.92 million years ago, respectively.

### 3.6 Expression profiles of *CsAKR* genes across tissues and under cold acclimation

To investigate the expression patterns of *CsAKR* genes, we analyzed transcriptome data across eight tissues and under cold acclimation. Genes with FPKM < 10 in all samples were excluded, resulting in 31 genes for tissue-specific analysis and 27 genes for cold acclimation analysis. The remaining genes were clustered into five distinct groups for each dataset (**Figures 5A, B**).

**Figure 5:**
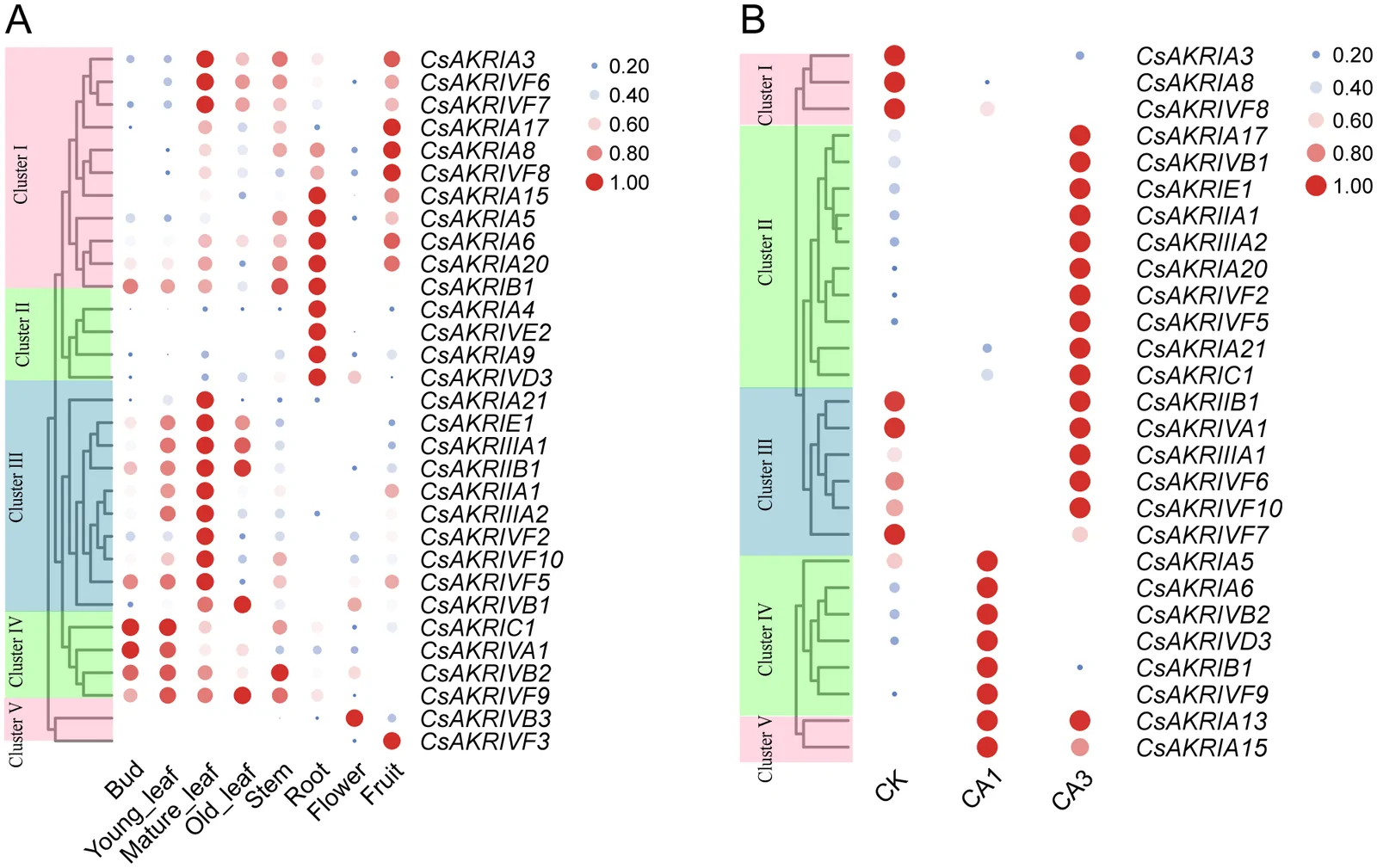
Expression profiles of *CsAKR* genes across different tissues and under cold acclimation. FPKM values were log_2_(FPKM + 1)-transformed and subjected to Z-score normalization by rows before clustering. Genes with FPKM < 10 in all samples were excluded; 31 genes were retained for tissue-specific analysis and 27 genes for cold acclimation analysis. **(A)** Heatmap showing tissue-specific expression patterns of *CsAKR* genes across eight tissues. **(B)** Heatmap showing expression patterns of *CsAKR* genes under non-acclimated (CK), fully acclimated (CA1) and de-acclimation (CA3).

In tissue-specific expression (**Figure 5A**; **Supplementary Table S7A**), Cluster I was predominantly expressed in mature leaves and fruits, with *CsAKRIVF6* exhibiting the highest transcript abundance in mature leaves. However, some members within this cluster, including *CsAKRIA5*, *CsAKRIA6*, and *CsAKRIB1*, also showed high expression levels in roots. Cluster III displayed leaf-enhanced expression, with higher transcript levels in mature/old leaves but low expression in roots and flowers. Cluster IV exhibited constitutive expression across most tissues at moderate levels, including *CsAKRIVA1*, *CsAKRIVB2*, *CsAKRIVF9*, and *CsAKRIC1.* Cluster V showed flower- or fruit-preferential expression.

Under cold acclimation (**Figure 5B**; **Supplementary Table S7B**), Cluster I was cold-repressed and its expression remained low upon de-acclimation. Cluster II showed de-acclimation-induced upregulation, including *CsAKRIVB1*. Cluster III displayed a cold-repressed pattern that was reversed by de-acclimation, with *CsAKRIVF6* and *CsAKRIVF7* markedly reduced during cold acclimation and rebounding upon de-acclimation; *CsAKRIIB1* also belonged to this cluster. Cluster IV exhibited a cold-induced, de-acclimation-repressed pattern. Among them, *CsAKRIVB2* showed the most pronounced induction, while *CsAKRIVF9* showed only a modest increase, suggesting a weaker cold response. Cluster V showed cold-induced and sustained expression.

Under salt and drought stresses, *CsAKRIVB2* also showed responsiveness (**Supplementary Figure S2A, B**, **Supplementary Tables S7C, D**). Based on its cold-inducible and sustained expression pattern, promoter features, and multi-stress responsiveness, *CsAKRIVB2* was selected for functional characterization.

### 3.7 RT-qPCR validation of candidate *CsAKR* genes under cold stress

To validate the transcriptome-based expression patterns, we selected 12 cold-responsive *CsAKR* genes for RT-qPCR analysis under cold stress (4 °C, 0–48 h). The selected genes were distributed across Clusters I, III, IV, and V and showed a response from CK(non-acclimated) to CA1(fully acclimated). The selected genes showed diverse expression trends that largely corroborated the transcriptome data (**Figure 6**). Among them, *CsAKRIVB2* displayed the most pronounced and rapid cold induction. Relative to the 0-h time point, its expression increased approximately 16-fold at 4 h, peaked at 24.8-fold at 12 h, and remained at 16.6-fold at 48 h. This rapid induction followed by sustained elevation was unique among all candidates tested. Other genes showed distinct trends: *CsAKRIA3* and *CsAKRIIIA1* were continuously repressed, whereas *CsAKRIVF6* and *CsAKRIVF7* exhibited an initial decline followed by recovery, but none matched the amplitude or persistence of *CsAKRIVB2* induction.

**Figure 6:**
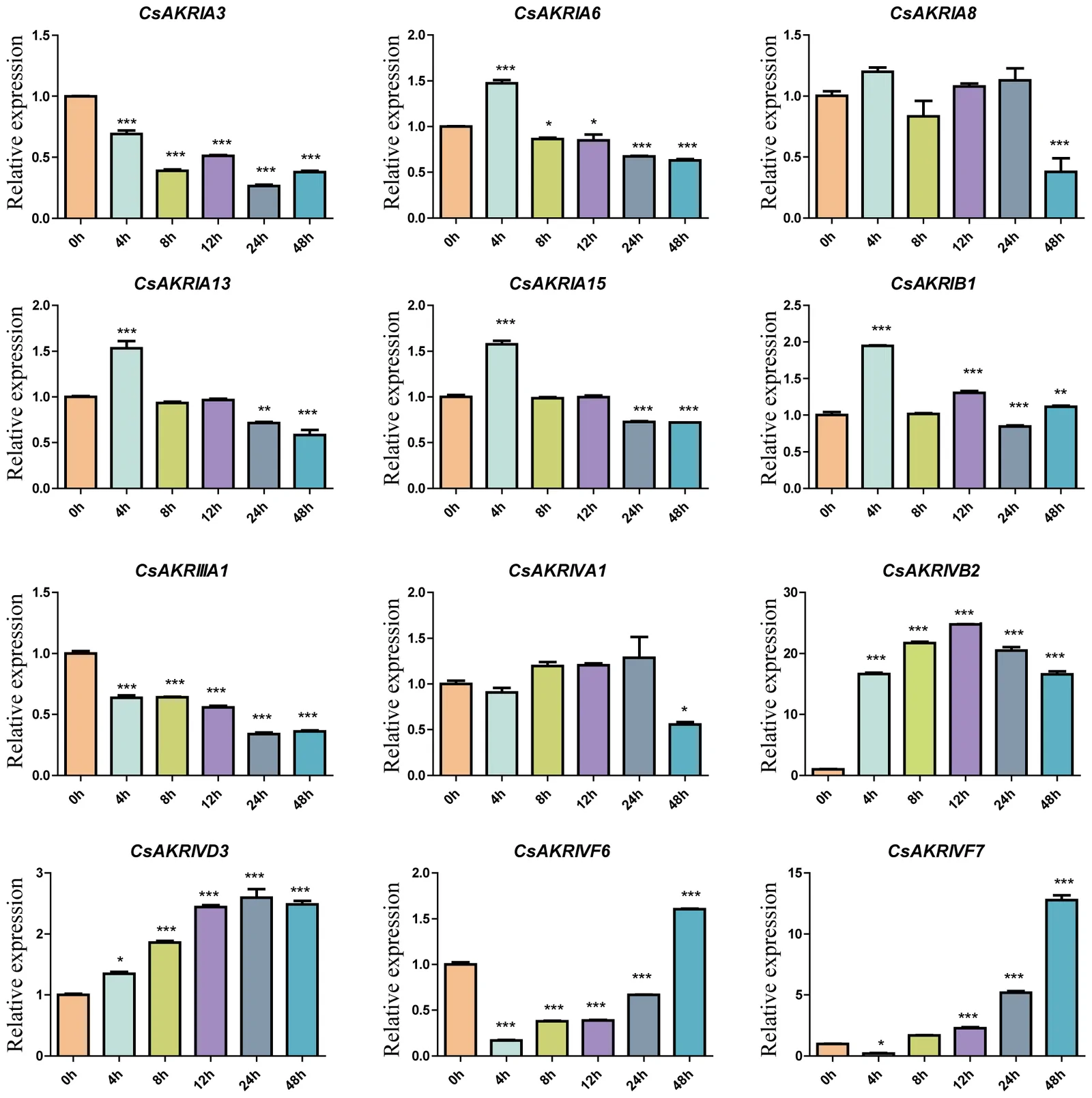
RT-qPCR validation of candidate *CsAKR* genes under cold stress. Relative expression levels of 12 *CsAKR* genes in tea plant leaves under cold stress (4 °C) at 0, 4, 8, 12, 24, and 48 h. Expression was normalized to *CsActin* and calculated using the 2^-ΔΔCt^ method. Data are shown as mean ± SD (n = 3). Asterisks indicate significant differences compared with the 0 h control (*P < 0.05, **P < 0.01, ***P < 0.001; Student’s t-test).

### 3.8 Selection of *CsAKRIVB2* for functional characterization

To prioritize candidates for functional analysis, we integrated transcriptomic profiling, RT-qPCR validation, and promoter architecture analysis. Among the cold-responsive *CsAKR* genes, *CsAKRIVB2* exhibited several distinctive features. First, it showed the most pronounced and sustained cold induction among all family members in both transcriptome (**Figure 5B**) and RT-qPCR data (**Figure 6**), with 16-fold induction at 4 h, peaking at 24.8-fold at 12 h and remaining 16.6-fold elevated at 48 h. Second, its promoter harbors seven ARE motifs, the highest counts among all 48 *CsAKR* genes (**Figure 4**; **Supplementary Table S5**). Third, *CsAKRIVB2* belongs to the IVB subfamily, which is closely related to the *Arabidopsis* detoxification enzymes *AKR4C8* and *AKR4C9* (Simpson et al., 2009). Fourth, *CsAKRIVB2* responded not only to cold but also to salt and drought stresses (**Supplementary Figure S2**), indicating broad-spectrum stress responsiveness. Although *CsAKRIVF9* also showed cold-induced expression and its promoter contains an LTR element, its induction amplitude was lower than that of *CsAKRIVB2*, and its promoter contained fewer ARE motifs (two versus seven). Based on this integrated assessment, *CsAKRIVB2* was selected as the most promising candidate for further functional characterization.

### 3.9 Subcellular localization of *CsAKRIVB2*

To determine the subcellular localization of *CsAKRIVB2*, we transiently expressed the 35S::*CsAKRIVB2*-*GFP* fusion construct and the 35S::*GFP* control in *Nicotiana benthamiana* leaf epidermal cells. Confocal microscopy showed that the free *GFP* control distributed uniformly throughout the nucleus and cytoplasm (**Figure 7A**). Similar to 35S::*GFP*, 35S::*CsAKRIVB2*-*GFP* was detected in both compartments (**Figure 7B**). Co-localization with the mCherry nuclear marker confirmed the nuclear presence of *CsAKRIVB2*-*GFP*, with overlapping GFP and mCherry signals in the merged images. DAPI staining independently confirmed the nuclear localization of *CsAKRIVB2*-*GFP* (**Supplementary Figure S3**). These results indicate that *CsAKRIVB2* is a nucleocytoplasmic protein, suggesting potential roles in both the cytoplasm and the nucleus.

**Figure 7:**
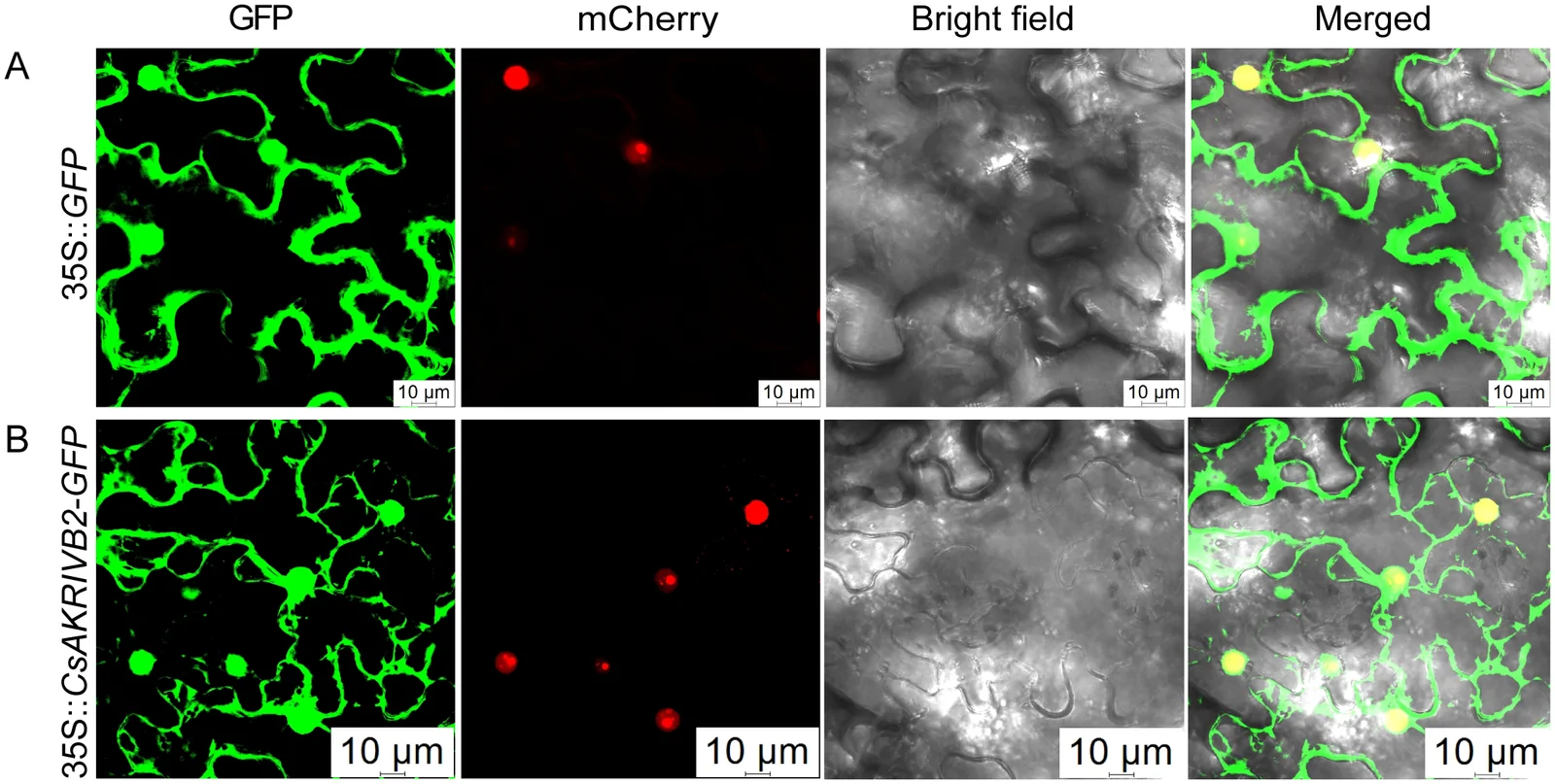
Subcellular localization of *CsAKRIVB2* in *Nicotiana benthamiana* leaf epidermal cells. **(A)** Confocal images of 35S::*GFP* (control) showing GFP fluorescence in the nucleus and cytoplasm. **(B)** Confocal images of 35S::*CsAKRIVB2*-*GFP* showing GFP fluorescence in both the nucleus and the cytoplasm. From left to right: GFP fluorescence, mCherry (nuclear marker), bright field, and merged images. Scale bars = 10 μm.

### 3.10 Heterologous expression of *CsAKRIVB2* enhances cold tolerance and reduces ROS accumulation

To experimentally verify the role of *CsAKRIVB2* in cold stress tolerance, we first examined its function through heterologous expression in *E. coli* and transient overexpression in *N. benthamiana* leaves. Heterologous expression of *CsAKRIVB2* significantly enhanced the cold tolerance of recombinant *E. coli* cells, as shown by improved survival in spot assays and increased growth rates at low temperature (**Supplementary Figure S4**).

In *N. benthamiana* leaves transiently overexpressing *CsAKRIVB2*, NBT and DAB staining showed no difference between the 35S::00 empty vector control and 35S::*CsAKRIVB2* under normal growth conditions. Under cold treatment (4 °C, 4 h), however, the 35S::*CsAKRIVB2* leaves exhibited significantly lighter staining than the 35S::00 control (**Figure 8**). NBT staining revealed sparse blue spots in 35S::*CsAKRIVB2* leaves, in contrast to the large, intense blue patches in control leaves, indicating reduced superoxide anion (O_2_^-^) accumulation. DAB staining showed pale brown coloration in 35S::*CsAKRIVB2* leaves, compared with dark brown precipitates in controls, reflecting decreased hydrogen peroxide (H_2_O_2_) levels. These results demonstrate that transient overexpression of *CsAKRIVB2* alleviates cold-induced ROS accumulation in plant cells.

**Figure 8:**
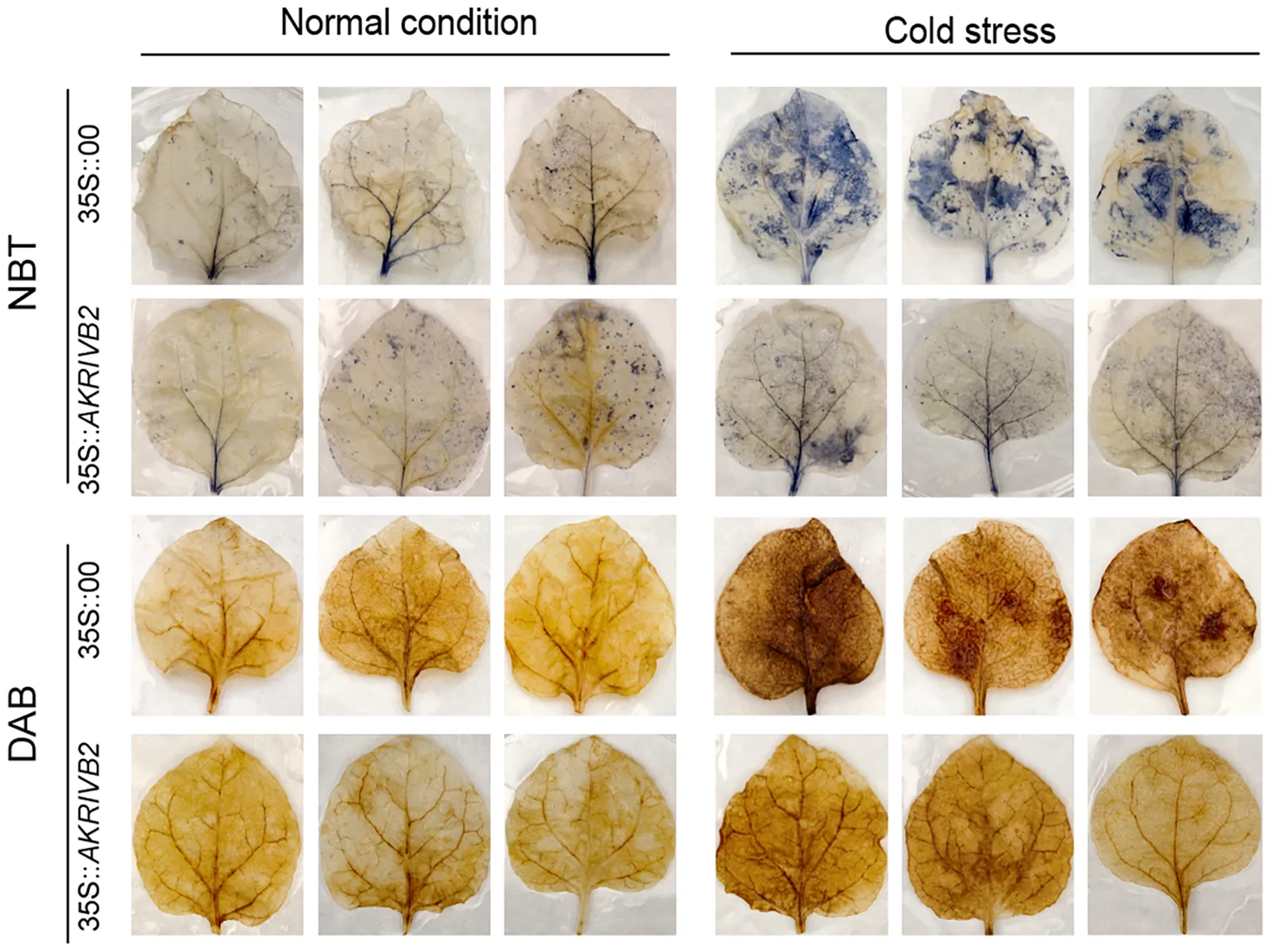
Transient overexpression of *CsAKRIVB2* reduces ROS accumulation in *Nicotiana benthamiana* leaves under cold stress. NBT and DAB staining of *N. benthamiana* leaves transiently expressing 35S::00 (empty vector control) or 35S::*CsAKRIVB2* under normal and cold treatment (4 °C, 4 h) conditions.

### 3.11 *CsAKRIVB2* overexpression enhances cold tolerance and alleviates oxidative damage in transgenic *A. thaliana*

To further validate the function of *CsAKRIVB2* in a stable genetic system, we generated transgenic *A. thaliana* lines constitutively overexpressing *CsAKRIVB2*. Six T_3_ homozygous lines were obtained, and RT-qPCR confirmed *CsAKRIVB2* transcript accumulation in all transgenic lines, whereas no specific amplification was detected in WT plants (Ct > 35 cycles). Among the six lines, OE1 and OE9 were selected as representative lines with moderate and high transgene expression levels, respectively, for detailed characterization (**Supplementary Figure S5A**).

Under normal growth, no phenotypic differences were observed among WT, OE1, and OE9. Following freezing treatment (–20 °C, 2 h) and 24 h recovery, however, OE1 and OE9 exhibited markedly better growth than WT (**Figure 9A**), with survival rates of 70.8% and 79.2%, respectively, compared with 41.7% for WT (**Figure 9C**; **Supplementary Table S10**).

**Figure 9:**
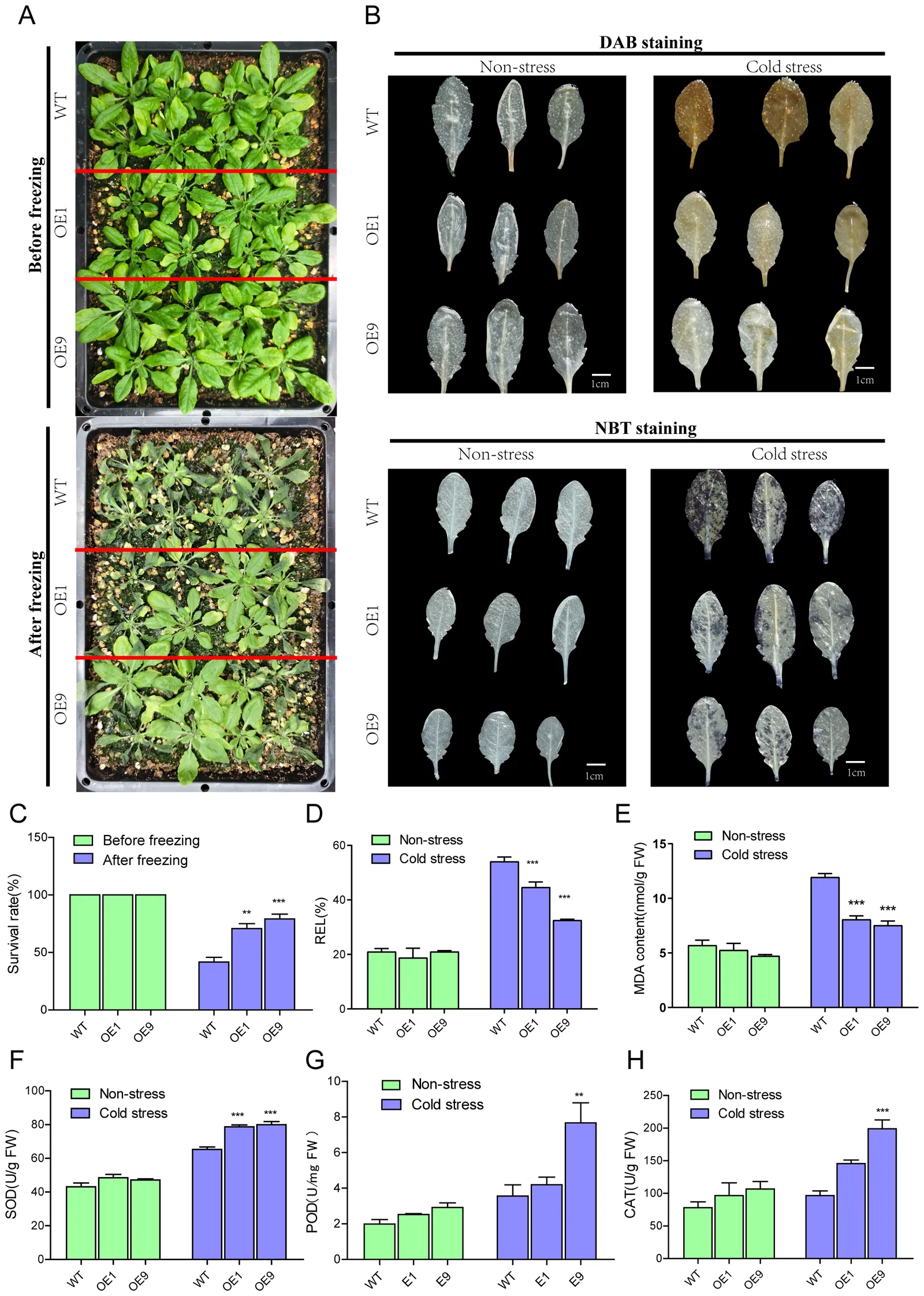
*CsAKRIVB2* overexpression enhances cold tolerance and alleviates oxidative damage in transgenic *A. thaliana*. **(A)** Phenotypes of WT, OE1, and OE9 plants under normal conditions and after freezing treatment (-20 °C, 2 h) followed by 24 h recovery. **(B)** DAB and NBT staining of leaves under normal and cold treatment (4 °C, 4 h) for detection of H_2_O_2_ and O_2_^-^ accumulation, respectively. **(C)** Survival rates after freezing treatment. **(D)** Relative electrolyte leakage. **(E)** MDA content. **(F)** SOD activity. **(G)** POD activity. **(H)** CAT activity. Data in **(C–H)** are presented as mean ± SD (n = 3). Asterisks indicate significant differences compared with WT (*P < 0.05, **P < 0.01, ***P < 0.001; Student’s *t*-test).

Consistent with enhanced survival, OE lines showed reduced membrane damage under cold stress (4 °C, 4 h). Relative electrolyte leakage and malondialdehyde (MDA) content, an indicator of lipid peroxidation, were significantly lower in OE1 and OE9 than in WT (**Figures 9D, E**). DAB and NBT staining revealed less ROS accumulation in OE leaves under cold stress, with lighter staining intensity (**Figure 9B**). Quantitative measurements confirmed that H_2_O_2_ and O_2_^-^ contents in OE lines were significantly reduced (**Supplementary Figure S6**).

To examine the enzymatic basis of enhanced ROS scavenging, we measured the activities of three key antioxidant enzymes. Under normal conditions, no significant differences were detected among genotypes. After cold stress, SOD, POD, and CAT activities in OE1 and OE9 were all significantly higher than in WT (**Figures 9F–H**). Collectively, these results demonstrate that *CsAKRIVB2* overexpression enhances cold tolerance by strengthening the antioxidant enzyme system, thereby reducing ROS accumulation and alleviating oxidative membrane damage.

### 3.12 *CsAKRIVB2* overexpression is associated with activation of the *CBF*-*COR* cold signaling pathway and upregulation of antioxidant enzyme-encoding genes

To elucidate the molecular mechanisms underlying the enhanced cold tolerance, we examined the expression of key genes in the *CBF*-*COR* (C-repeat Binding Factor/Cold-Regulated) cold signaling pathway and the antioxidant defense system by RT-qPCR.

Under cold stress (4 °C), *CBF1*, *CBF2*, and *CBF3* were strongly induced at 4 h in both WT and OE lines, reaching 12.5-, 10.6-, and 12.8-fold increases in WT, respectively. By 24 h, their expression declined substantially in all genotypes, indicating that *CsAKRIVB2* overexpression did not alter the pulsatile activation pattern of CBF transcription factors (**Figure 10**). However, the downstream cold-regulated (COR) genes showed markedly enhanced and sustained activation in OE lines. *KIN1*, which dropped below the basal level in WT at 24 h, was upregulated to 4.7-fold and 9.1-fold of the WT level in OE1 and OE9, respectively. At 24 h, *RD29A* was induced 11.2-fold (OE1) and 4.9-fold (OE9) relative to WT, and *COR15A* was induced 1.4-fold in OE9. *COR15B* was also upregulated in OE1 at 24 h. *SnRK2.4*, encoding a kinase implicated in ABA-independent cold signaling, underwent severe cold-induced transcriptional repression in WT, with its transcript level plummeting to 3.8% of the basal level at 24 h. In OE1 and OE9, however, *SnRK2.4* expression was maintained at 9.7-fold and 7.1-fold of the WT level, respectively (**Figure 10**).

**Figure 10:**
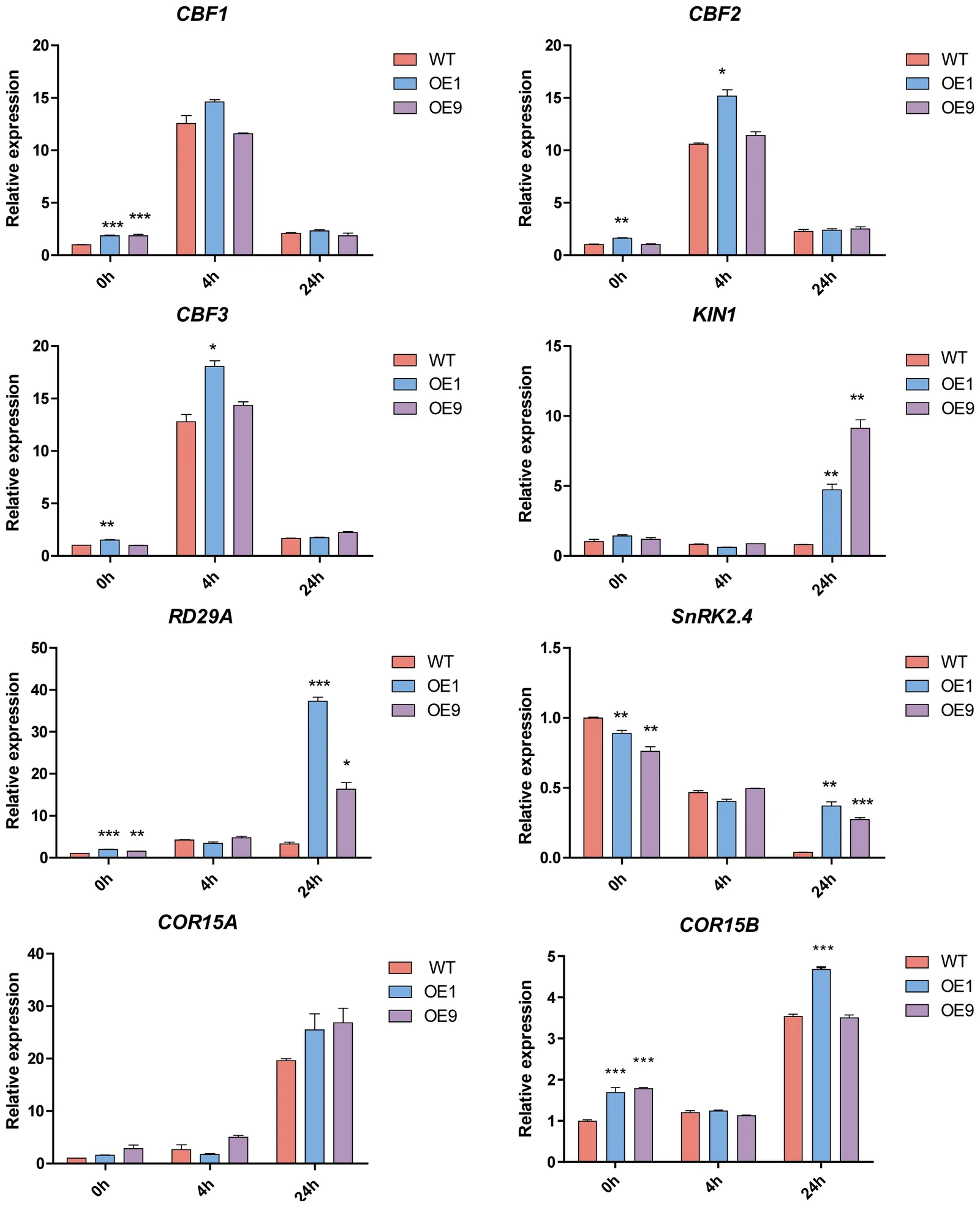
*CsAKRIVB2* overexpression activates the CBF-COR cold signaling pathway. Relative expression levels of *CBF1*, *CBF2*, *CBF3*, *KIN1*, *RD29A*, *SnRK2.4*, *COR15A*, and *COR15B* in WT, OE1, and OE9 lines at 0, 4, and 24 h under cold stress (4 °C). Transcript levels were normalized to *AtUBQ10* and expressed relative to the WT 0 h sample (set to 1.0). Data are shown as mean ± SD (n = 3 biological replicates). Asterisks indicate significant differences compared with WT (*P < 0.05, **P < 0.01, ***P < 0.001; Student’s t-test). Abbreviations: WT, wild type; OE, overexpressing; *CBF*, C-repeat binding factor; *KIN1*, cold and ABA inducible protein KIN1; *RD29A*, desiccation-responsive protein 29A; *SnRK2.4*,SNF1-related protein kinase 2.4; *COR15A*, cold-regulated protein 15A; *COR15B*, cold-regulated protein 15B.

At 4 h of cold stress, multiple antioxidant enzyme-encoding genes were significantly upregulated in OE lines relative to WT (**Figure 11**). *FSD1* (encoding Fe-superoxide dismutase) was upregulated to 3.7-fold and 1.8-fold of WT levels in OE1 and OE9, respectively. *CAT1* and *CAT2* were upregulated to 10.9-fold and 2.5-fold of WT levels in OE1, while *GR2* and *MDAR1* reached 2.1-fold and 5.2-fold, respectively. *APX2* showed exceptionally strong induction in OE1 at 4 h, reaching 357.5-fold of the WT level, and remained 13.6-fold higher at 24 h. These transcriptional changes aligned with the enhanced SOD, POD, and CAT enzyme activities observed in OE lines (**Figures 9H–J**), providing a molecular basis for the enhanced ROS-scavenging capacity.

**Figure 11:**
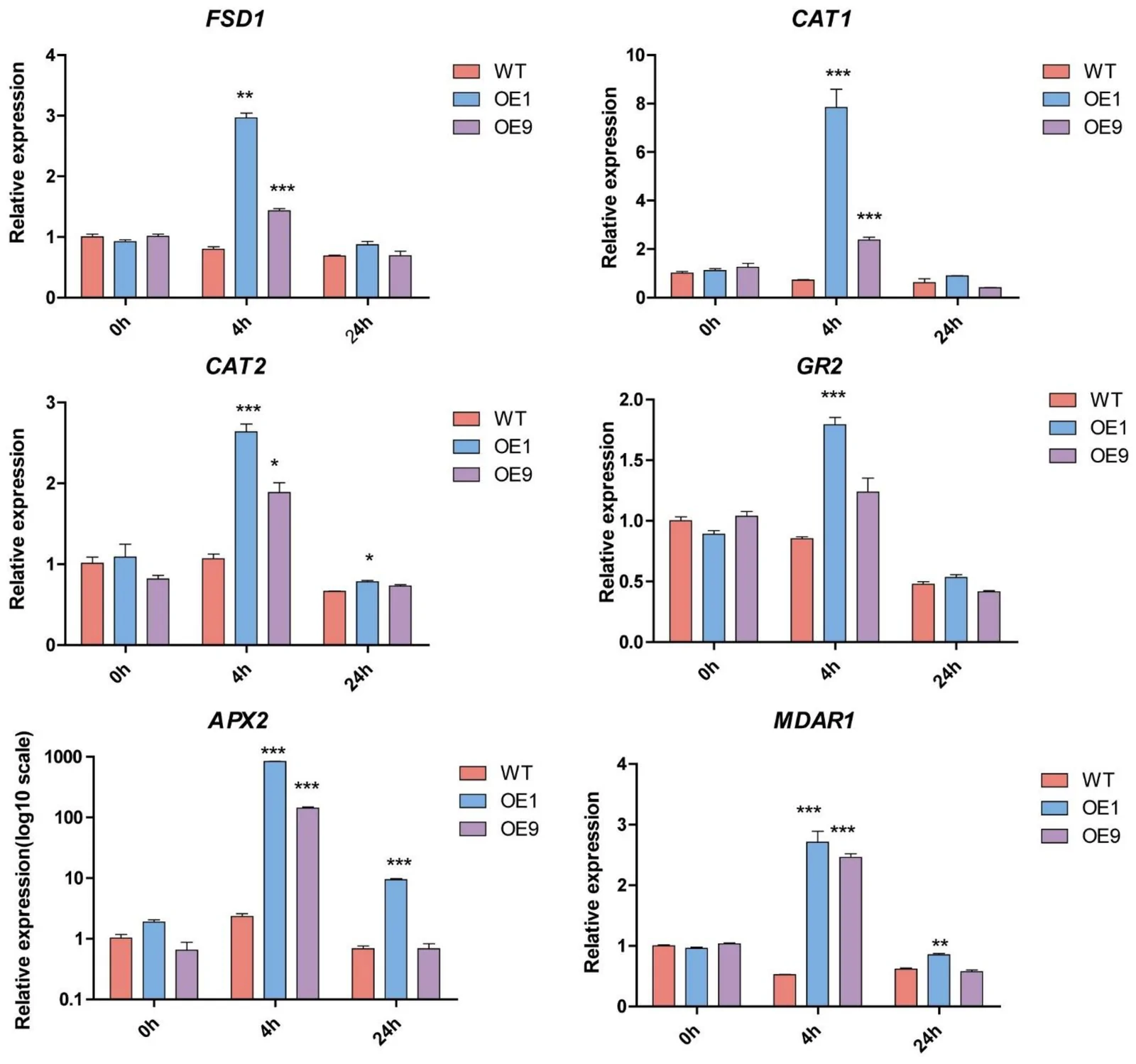
*CsAKRIVB2* overexpression upregulates antioxidant enzyme-encoding genes under cold stress. Relative expression levels of *FSD1*, *CAT1*, *CAT2*, *GR2*, *APX2*, and *MDAR1* in WT, OE1, and OE9 lines at 0, 4, and 24 h under cold stress (4 °C). Transcript levels were normalized to *AtUBQ10* and expressed relative to the WT 0 h sample (set to 1.0). Data are shown as mean ± SD (n = 3 biological replicates). Asterisks indicate significant differences compared with WT (*P < 0.05, **P < 0.01, ***P < 0.001; Student’s t-test).Abbreviations: WT, wild type; OE, overexpressing; *FSD1*, Fe-superoxide dismutase 1; *CAT*, catalase; *GR2*, glutathione reductase 2; *APX2*, ascorbate peroxidase 2; *MDAR1*, monodehydroascorbate reductase 1.

Collectively, these results demonstrate that *CsAKRIVB2* overexpression enhances cold tolerance, accompanied by reduced oxidative damage, increased antioxidant enzyme activities, and upregulated expression of multiple cold-responsive and antioxidant genes.

## 4 Discussion

We conducted the first systematic genome-wide identification of *AKR* genes in the tea plant, revealing 48 *CsAKR* members in four phylogenetic groups. Transcriptomic profiling, RT-qPCR, and functional assays identified *CsAKRIVB2* as a positive regulator of cold tolerance. The enhanced cold tolerance of *CsAKRIVB2*-overexpressing lines was accompanied by sustained *CBF*-*COR* pathway activity and a strengthened ROS-scavenging system, although the causal hierarchy among these phenotypes is not yet resolved.

The tea plant genome contains 48 *CsAKR* genes. This number is larger than the 22 in *Arabidopsis*, 28 in rice, 30 in *Medicago*, 28 in tomato, and 38 in sweet cherry (Yu et al., 2020; Guan et al., 2023; Guo et al., 2025). This suggests lineage-specific expansion in tea. As in other plants, Group I is the largest AKR subfamily (Krishnamurthy et al., 2022). In tea, Group I has 25 members. Phylogenetically, the *CsAKRIVB* subfamily forms a well-supported clade with *Arabidopsis AKR4C8* (*AT2G37760.2*) and *AKR4C9* (*AT2G37770.2*). These *Arabidopsis* proteins are aldehyde/ketone reductases with broad substrate specificity for cytotoxic carbonyls and are involved in stress-responsive detoxification (Simpson et al., 2009). Synteny analysis revealed five segmental duplication pairs. In each case, the duplicated genes lie on different chromosomes or at distal positions on the same chromosome, a typical pattern of segmental duplication (Wang et al., 2012). No tandemly duplicated *CsAKR* pairs were found. Thus, segmental duplication, not tandem duplication, drove the expansion of this family. This is consistent with the tea genome’s history of recurrent whole-genome duplications (Xia et al., 2017), which likely facilitated the retention and diversification of duplicated *AKR* genes. All orthologous *AKR* pairs between tea and *C. oleifera* or *C. chekiangoleosa* had *Ka/Ks* ratios below 1, indicating purifying selection. Their estimated divergence times match the evolutionary timeline of *Camellia* (Xia et al., 2017). The *CsAKRIVB1*/*CsAKRIVB2* pair has been retained under purifying selection, and the IVB subfamily occupies a conserved phylogenetic position. Together, these observations suggest that *CsAKRIVB2* is a strong candidate for functional study.

Stress-responsive *cis*-elements are widely distributed across *CsAKR* promoters. Notably, the *CsAKRIVB2* promoter lacks canonical LTR/DRE elements, indicating that its cold induction is unlikely to be mediated directly through the CBF/DREB–LTR pathway. Instead, the *CsAKRIVB2* promoter contains seven ARE motifs, the most among all 48 *CsAKR* genes (**Figure 4**; **Supplementary Table S5**).We note that PlantCARE annotates ARE as an anaerobic-responsive element (Lescot et al., 2002); however, ARE is also established as the antioxidant response element (core motif 5′-TGACnnnGC-3′) of the Nrf2/Keap1(nuclear factor erythroid 2-related factor 2/Kelch-like ECH-associated protein 1) pathway (Raghunath et al., 2018), and functional antioxidant response elements have been identified in the promoter of the aldo-keto reductase *AKR1B10* (Nishinaka et al., 2017).This high abundance may drive a feed-forward detoxification loop: cold-induced ROS activates ARE-binding transcription factors (e.g., TGA bZIP proteins), which are known to regulate redox-responsive genes (Garretón et al., 2002), thereby upregulating *CsAKRIVB2*. Consistent with this, *CsAKRIVB2* is induced not only by cold but also by salt and drought (**Supplementary Figure S2**), suggesting that its primary regulatory input is oxidative/carbonyl stress rather than a cold-specific signal. Dissecting the functional significance of these *cis*-elements through promoter deletion and mutagenesis will be a focus of our future work. Recent evidence highlights the importance of RCS detoxification: accumulation of reactive carbonyls, not ROS itself, is the primary cause of growth inhibition under abiotic stress (Sultana et al., 2024). The lower MDA and ROS levels in OE lines (**Figures 9B, E**) support this view. *CsAKRIVB2* localizes to both the nucleus and cytoplasm, as do other plant AKRs (Sengupta et al., 2015). This dual localization supports its two protective roles. In the cytoplasm, it detoxifies RCS from lipid peroxidation (Yalçinkaya et al., 2019). In the nucleus, it may scavenge genotoxic aldehydes that cross the nuclear membrane, thus protecting the transcriptional machinery from carbonyl damage (Winczura et al., 2012). Together, the ARE-rich promoter and the nucleocytoplasmic localization position *CsAKRIVB2* at the interface of oxidative/carbonyl stress sensing, detoxification, and the protection of transcriptional machinery under cold stress. Importantly, *CsAKRIVB2* is an NADPH-dependent oxidoreductase whose primary biochemical role is carbonyl detoxification. It is therefore unlikely to function as a direct transcriptional regulator, and the observed transcriptional changes in the OE lines are most parsimoniously interpreted as downstream consequences of its enzymatic activity—namely, the reduced carbonyl/oxidative stress—rather than as direct transcriptional regulatory functions of the protein itself.

An intriguing observation was the differential ROS accumulation between OE1 and OE9 under cold stress: OE9 accumulated higher H_2_O_2_ but lower O_2_^-^ than OE1 (**Supplementary Figure S6**). This pattern may be explained by the kinetic relationship between O_2_^-^ and H_2_O_2_ metabolism. O_2_^-^ is the primary ROS species generated under stress and is rapidly converted to H_2_O_2_ by SOD (Gill and Tuteja, 2010). In OE9, which exhibited higher SOD activity than OE1 (**Figure 9F**), the more efficient SOD-mediated dismutation of O_2_^-^ likely accounts for the lower O_2_^-^ accumulation (due to enhanced scavenging) and concurrently higher H_2_O_2_ levels (as a consequence of accelerated O_2_^-^ conversion). Both OE lines maintained H_2_O_2_ levels significantly below WT, indicating that H_2_O_2_ scavenging systems are also enhanced in both lines. Thus, the differential ROS pattern likely reflects a shift in flux through the O_2_^-^ -detoxifying versus H_2_O_2_ scavenging branches of the antioxidant network, rather than compromised ROS management in either line. Consistent with this interpretation, both OE1 and OE9 exhibited significantly improved physiological performance under cold stress compared with WT (**Figures 9C–E**).

*CsAKRIVB2*-overexpressing lines have lower electrolyte leakage and MDA content (**Figures 9D, E**), indicating that *CsAKRIVB2* alleviates cold-induced membrane lipid peroxidation. MDA is not just a biomarker. It is a cytotoxic aldehyde that forms covalent adducts with proteins and nucleic acids, worsening cell damage (Mano, 2012). Detoxifying MDA thus prevents secondary carbonyl toxicity. SOD, POD, and CAT activities increase (**Figures 9F–H**) while ROS levels drop (**Figures 9B, D, E**). This may partly be attributable to changes in RCS-mediated regulation of these enzyme activities. Reactive aldehydes like HNE and MDA can covalently modify and inactivate antioxidant enzymes by carbonylating key amino acid residues (Sengupta et al., 2015). For example, methylglyoxal, an RCS detoxified by AKRs, directly inhibits cytosolic ascorbate peroxidase through covalent modification (Hoque et al., 2012). By scavenging these aldehydes, *CsAKRIVB2* may help keep these enzymes active. *OE* lines also show higher transcript levels of genes encoding antioxidant enzymes (**Figure 11**). *APX2* transcript increases 357.5-fold over the wild type. *APX2* encodes a cytosolic ascorbate peroxidase and is a key stress-responsive isoform in *Arabidopsis* (Panchuk et al., 2002; Fryer et al., 2003). In tomato, *SlAPX2* is positively regulated by ABA and enhances tolerance to heat and cold (Liang et al., 2025), indicating that *APX2* plays a conserved role in stress tolerance across species. This strong upregulation likely explains the lower H_2_O_2_ levels (**Figure 9B**) and higher POD activity (**Figure 9G**) in OE lines. *CAT1*, *FSD1*, *MDAR1*, and *GR2* are also upregulated along with *APX2*. This suggests that *CsAKRIVB2* activates antioxidant gene transcription via a redox-sensitive feedback loop, possibly through changes in H_2_O_2_ levels or the NADPH/NADP^+^ ratio caused by AKR activity. Thus, *CsAKRIVB2* boosts antioxidant defense both by protecting enzyme activity post-translationally and by amplifying the transcription of antioxidant genes.

In *CsAKRIVB2* OE lines, the transcript levels of downstream cold-responsive genes, including *COR15A*, *COR15B*, *RD29A*, and *KIN1*, were sustained at higher levels under prolonged cold stress, whereas the transient induction of *CBF1–3* at 4 h was comparable between WT and OE lines (**Figure 10**). This observation suggests that *CsAKRIVB2* overexpression does not affect the initial activation of CBF transcription factors but rather helps maintain the downstream output of the *CBF*-*COR* pathway during extended cold exposure. The *ICE1*-*CBF*-*COR* cascade is a well-established regulator of cold acclimation (Shi et al., 2018), and *COR* genes encode cryoprotective proteins that improve freezing tolerance (Thomashow, 1999). The sustained expression of these COR genes in OE lines is consistent with the reduced electrolyte leakage and improved survival rates observed under freezing conditions (**Figures 9C, D**). We speculate that this effect may be attributed to the reduced carbonyl and oxidative stress in OE lines (**Figures 9E–G**), which could preserve the transcriptional machinery and cellular environment required for sustained COR gene expression, consistent with the nucleocytoplasmic localization of *CsAKRIVB2* discussed above. However, the precise molecular link between *CsAKRIVB2*-mediated detoxification and COR gene expression remains to be elucidated.

## 5 Conclusion

This study provides the first genome-wide characterization of the aldo-keto reductase (AKR) gene family in *Camellia sinensis*. We identified 48 *CsAKR* genes, which fall into four phylogenetic groups. Evolutionary analyses showed that segmental duplication drove the expansion of this family, and all retained duplicates evolved under purifying selection. Among the family members, *CsAKRIVB2* stood out: its promoter contains the highest number of ARE motifs, and its expression is strongly induced by cold. We tested its function through heterologous expression in *E. coli*, transient overexpression in *N. benthamiana*, and stable overexpression in transgenic *A. thaliana*. Overexpression of *CsAKRIVB2* reduced cold-induced membrane lipid peroxidation (lower MDA content and electrolyte leakage), increased the activities of SOD, POD, and CAT, and boosted the transcript levels of antioxidant genes such as *APX2*, *CAT1*, and *FSD1*. In addition, under prolonged cold stress *CsAKRIVB2* overexpression was associated with sustained *CBF*-*COR* cold signaling and the maintained expression of multiple cold-responsive genes, tentatively linking AKR-mediated detoxification to CBF-dependent cold signaling. *CsAKRIVB2* is therefore a promising candidate for breeding cold-tolerant tea varieties, although the precise causal relationship between its detoxification activity and cold-responsive gene expression requires further validation. Future work should focus on three aspects: whether and how *CsAKRIVB2* activity contributes to the sustained cold-responsive gene expression, the protein interaction network of *CsAKRIVB2*, and functional validation in tea plants using genetic approaches.

## Data Availability

The original contributions presented in the study are included in the article/**Supplementary Material**. Further inquiries can be directed to the corresponding author.
