# Supplementary material for: Genome-wide identification of the aldo-keto reductase gene family and functional characterization of CsAKRIVB2 conferring cold tolerance in tea plant (Camellia sinensis)
Source: Front Plant Sci. 2026 Sep 24;17:1950368. doi: 10.3389/fpls.2026.1950368 (PMC13645551; doi:10.3389/fpls.2026.1950368)
Supplement: Supplementary file 1 [file DataSheet1.pdf]

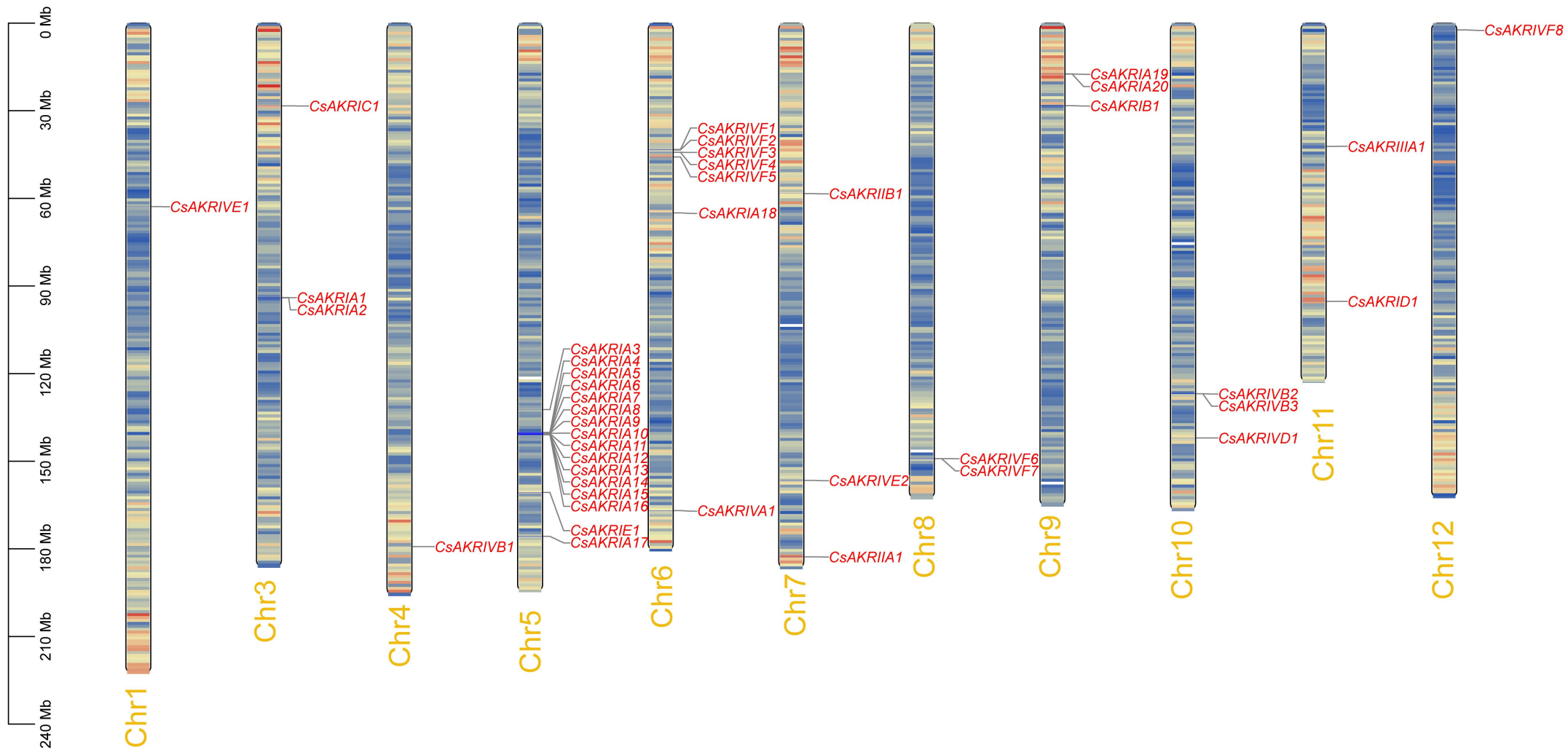

Figure S1. Chromosomal distribution of *CsAKR* genes.

Genomic locations of 48 *CsAKR* genes on the 15 *Camellia sinensis* chromosomes. Six genes localized on unassembled contigs are not shown. Chromosome numbers are indicated at the top of each bar. The scale bar represents chromosome length in megabases (Mb).

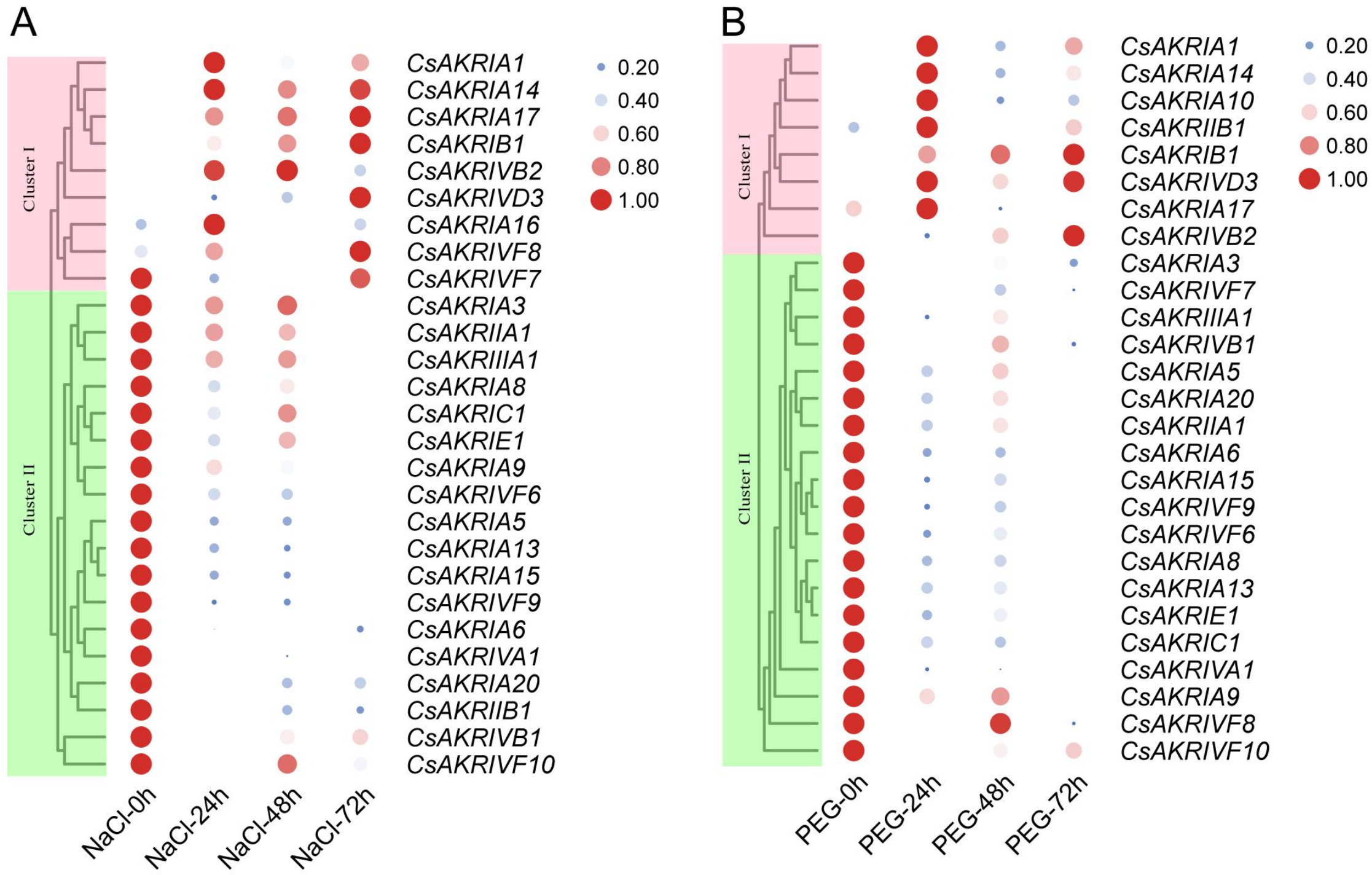

Figure S2. Expression patterns of *CsAKR* genes under salt and drought stresses.

FPKM values were $\log_2$(FPKM + 1)-transformed and subjected to Z-score normalization by rows before clustering.Genes with FPKM < 10 in all samples were excluded. (A) Heatmap showing expression patterns under salt stress (200 mM NaCl) at multiple time points. (B) Heatmap showing expression patterns under PEG-simulated drought stress at multiple time points.

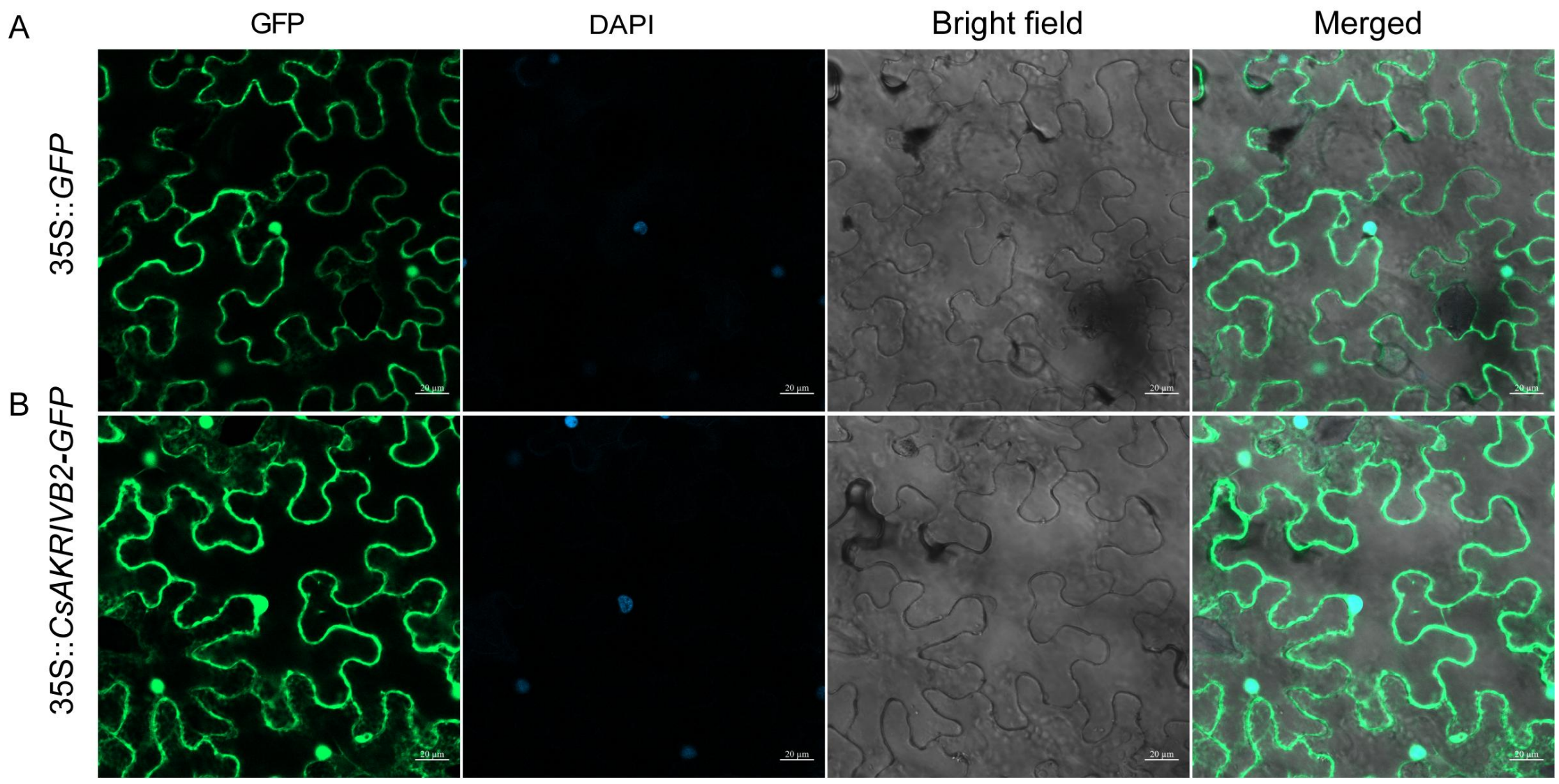

Figure S3. DAPI staining confirms the nuclear localization of CsAKRIVB2.

Confocal images of 35S::*CsAKRIVB2-GFP* in *Nicotiana benthamiana* leaf epidermal cells, showing *GFP* fluorescence (green), DAPI staining (blue), bright field, and merged images. Overlap of *GFP* and DAPI signals in the merged image confirms nuclear localization. Scale bar = 20 μm.

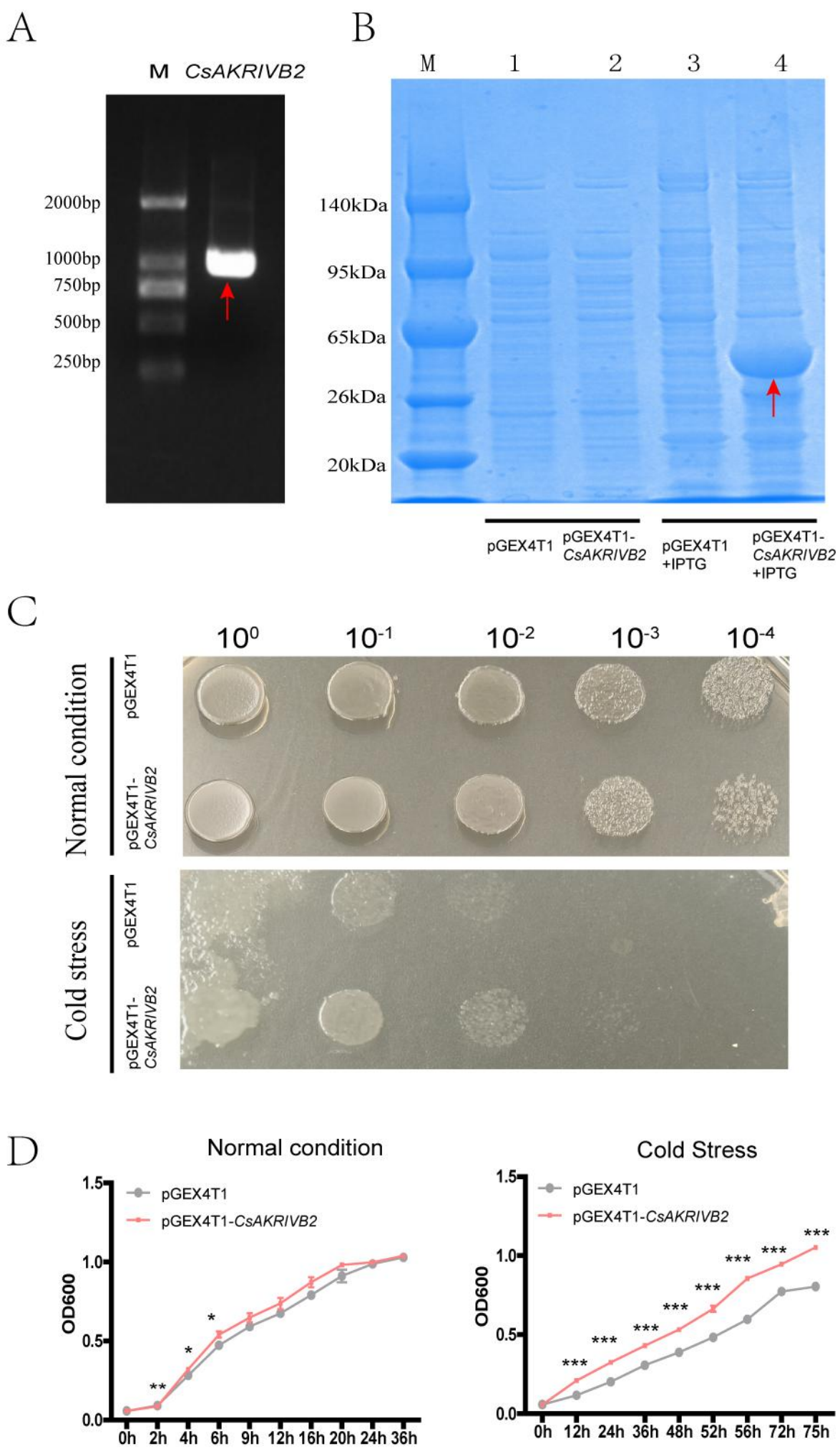

Figure S4. Molecular cloning, prokaryotic expression, and cold tolerance assays of *CsAKRIVB2* in *Escherichia coli*.

(A) Agarose gel electrophoresis of the PCR- amplified *CsAKRIVB2* coding sequence (948 bp). (B) SDS-PAGE analysis of the recombinant GST-*CsAKRIVB2* fusion protein (~61 kDa) expressed in *E. coli* after 0.5 mM IPTG induction. (C) Spot assay of *E. coli* cells harboring pGEX-4T-1 (empty vector) or pGEX-4T-1-*CsAKRIVB2* under normal conditions and after freezing treatment (–20°C, 12 h). (D) Growth curves (OD_{600}) at 15°C and 37°C. Data are shown as mean ± SD (n = 6). Asterisks indicate significant differences (*P < 0.05, **P < 0.01, ***P < 0.001; Student's t-test).

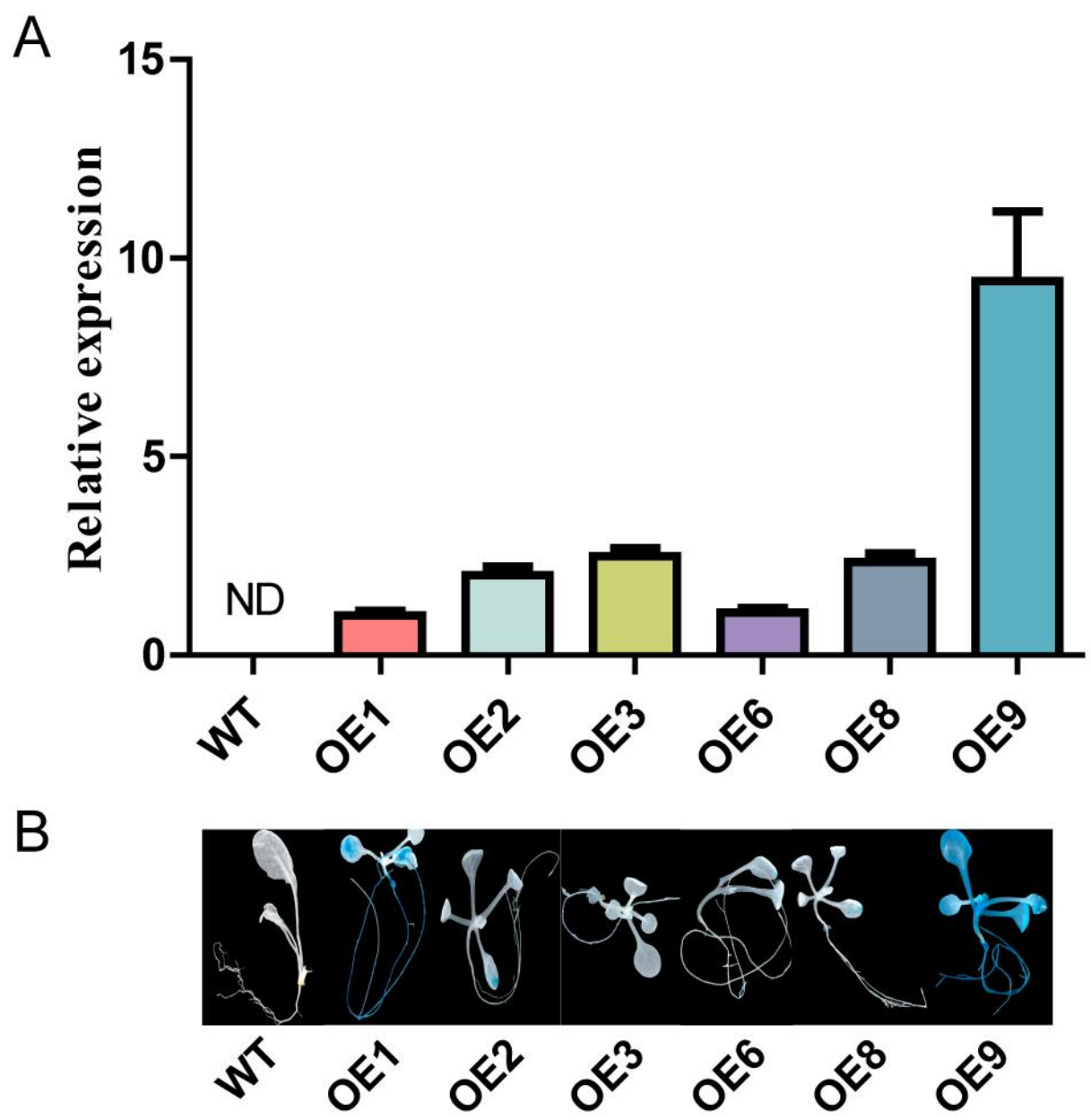

Figure S5. Molecular characterization of transgenic *Arabidopsis* lines overexpressing *CsAKRIVB2*.

(A) Relative expression levels of *CsAKRIVB2* in six T3 homozygous lines by RT- qPCR. *CsAKRIVB2* transcripts were detected in all transgenic lines, while no specific amplification was observed in WT plants (Ct > 35 cycles). Expression levels in transgenic lines are shown relative to OE1 (set to 1.0). Data are mean ± SD (n = 3). (B) GUS staining (qualitative confirmation of transgene integration) of 10-day-old T3 seedlings. OE1 and OE9 were selected for further analysis.

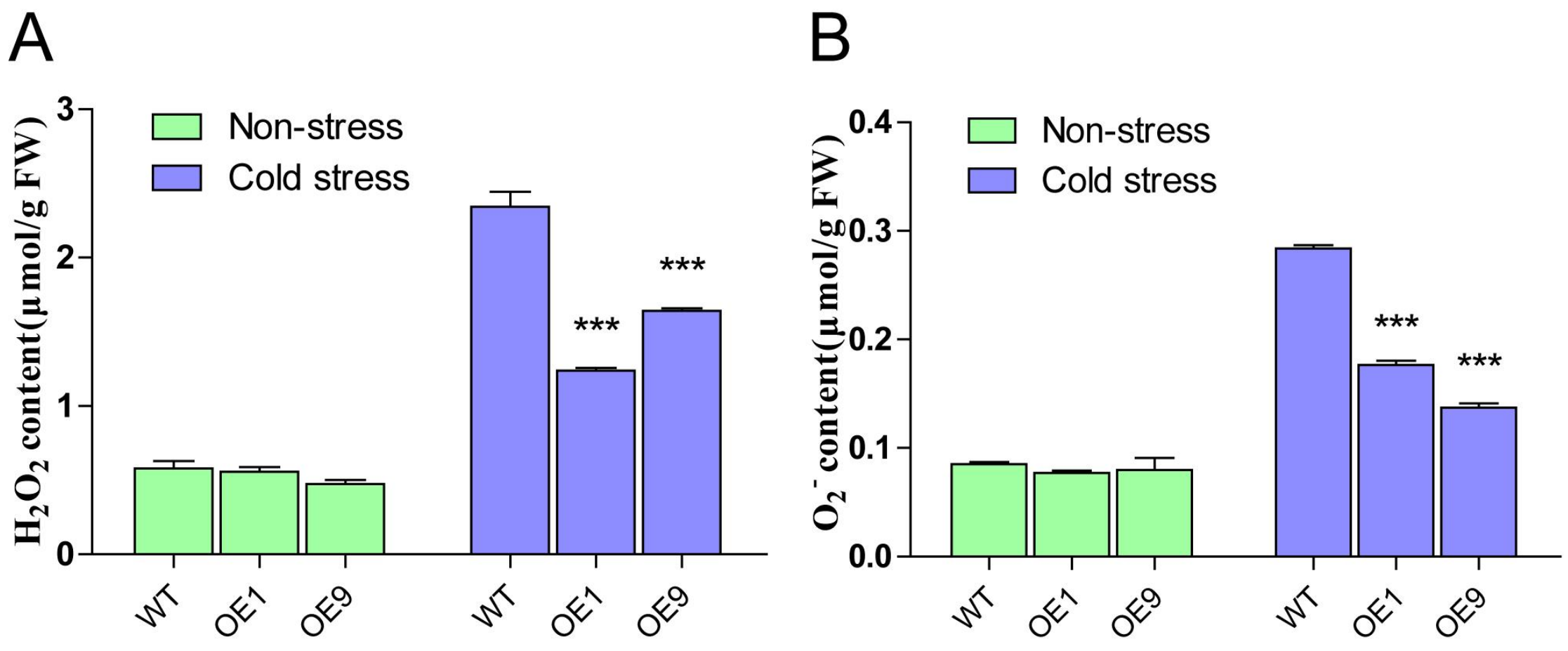

Figure S6. Quantification of ROS accumulation in WT, OE1, and OE9 lines under cold stress. (A) H_2O_2 content. (B) O_2^- content. Data are shown as mean ± SD (n = 3). Asterisks indicate significant differences compared with WT (P < 0.05, *P < 0.01, **P < 0.001; Student's *t*-test).
